# Hind-Casting the Quantity and Composition of Discards by Mixed Demersal Fisheries in the North Sea

**DOI:** 10.1371/journal.pone.0117078

**Published:** 2015-03-16

**Authors:** Michael R. Heath, Robin M. Cook

**Affiliations:** Department of Mathematics and Statistics, University of Strathclyde, Glasgow, Scotland, United Kingdom; Griffith University, AUSTRALIA

## Abstract

Many commercial fisheries seek to maximise the economic value of the catch that they bring ashore and market for human consumption by discarding undersize or low value fish. Information on the quantity, size and species composition of discarded fish is vital for stock assessments and for devising legislation to minimise the practice. However, except for a few major species, data are usually extremely sparse and reliant on observers aboard a small sample of fishing vessels. Expanding these data to estimate total regional discards is highly problematic. Here, we develop a method for utilising additional information from scientific trawl surveys to model the quantities of fish discarded by the commercial fisheries. As a case-study, we apply the model to the North Sea over the period 1978-2011, and show a long-term decline in the overall quantity of fish discarded, but an increase in the proportion of catch which is thrown away. The composition of discarded catch has shifted from predominantly (∼80%) roundfish, to >50% flatfish. Undersized plaice constitute the largest single fraction of discards, unchanged from the beginning of the 20th century. Overall, around 60% of discarded fish are rejected on the basis of size rather than for reasons of species value or quota restrictions. The analysis shows that much more information can be gained on discarding by utilising additional sources of data rather than relying solely on information gathered by observers. In addition, it is clear that reducing fishing intensity and rebuilding stocks is likely to be more effective at reducing discards in the long term, than any technical legislation to outlaw the practice in the short term.

## Introduction

Most commercial fisheries supplying fish for human consumption will seek to capture species which are economically valuable and in demand by consumers. However, the catch often includes fish, benthos, macrophytes, and occasionally marine mammals and seabirds which are unsuitable for consumption and/or have little or no market value. Usually, this material is sorted from the catch and discarded at sea [1–6]. Regarding the fish components of the catch, modern-day discarding decisions made by fishers depend on a complex interaction between legal and practical constraints and economic trade-offs [7]. Legal issues include minimum landing sizes and quota restrictions on quantities of each species that can be landed, the extent of enforcement, and the penalties for infringement. Practical constraints are factors such as the amount of catch that can be accommodated aboard the vessel, whilst economic trade-offs include market values of each species relative to their abundance in catches [2,8–12]. However, regardless of the reason for discarding, the majority of rejected fish are already dead or fatally damaged as a result of the capture process, adding unintended mortality to both target and non-target species. For this reason discarding is widely regarded as being wasteful and contrary to ethical principles of exploiting living resources [13–16].

Information on quantities of fish which are discarded is important for a variety of reasons. First, if discards constitute more than a trivial fraction of catches, then stock assessments based solely on landed quantities of fish will result in biased estimates of mortality rate and stock biomass, in turn leading to erroneous perceptions of sustainable exploitation rates [17–20]. Secondly, devising regulations to curtail discarding by, for example, compensating for the imposition a landing obligation by inflating landing quotas so as to accommodate some or all of the fish otherwise discarded (so called “quota-uplift”) [21,22], requires good knowledge of contemporary discarding practices so that precise forecasts of catches can be made. This is one of the options proposed in reforms of the EU Common Fisheries Policy (CFP) [22,23–27]. Finally, the biomass embodied in discarded fish is not lost to the ecosystem, but constitutes a potentially important food source for scavenging and opportunistic-feeding species including other fish, benthos, birds and mammals [28–39]. The ecosystem impacts of regulations to reduce discards cannot be determined in the absence of comprehensive information on quantities involved [14,40,41].

Monitoring of the quantities of fish landed at ports is, in principle, a straightforward task since the fish are generally sorted and weighed prior to sale. However, gathering information on the quantity and composition of fish discarded at sea is extremely demanding, requiring observers aboard individual vessels to collect data without interrupting the normal fishing operations [42–45]. Some administrations require that an observer be carried on every vessel operating in a fishery to monitor catch and discards, but this is the exception rather than the norm worldwide. More typically, observers can be placed on only a small proportion of vessels and fishing trips. In addition, the cooperation of vessel skippers and crew may be voluntary, and fishers may modify their behaviour in the presence of observers, leading to potential bias in the observed discarding rates [46,47].

Expanding data from observer samples, which are thinly spread across a range of fishing methods, vessel sizes, regions and times of year, to produce regional estimates of total quantities of fish discarded, represents a formidable statistical challenge. There are a number of approaches [48–58], but most rely on estimating the mean proportion (*p*) of catch weight which is discarded in the sampled fraction of the fleet. Then, assuming that this proportion applies uniformly across the fleet as a whole, and given the fleet total weight of fish landed (*L*), the total weight discarded (*D*) is given by *D* = *Lp/*(1—*p*). One of the problems with this approach is that as *p* approaches 1, the scaling term *p/*(1—*p*) approaches infinity so that for heavily discarded species the estimates of *D* are highly sensitive to uncertainty in *p*. Clearly this makes the estimation of total quantities discarded problematic.

In the North Sea, where observer-based fisheries sampling has been carried out by some nations for more than 30 years, credible time-series of species-specific, regional-scale annual discards integrated across the full range of fleets, have been compiled only for the five major targeted demersal species (cod, haddock, whiting, plaice and sole [59]). Data on all the other species captured by fisheries in the North Sea are largely insufficient or inaccessible for the purposes of estimating annual weights discarded for years prior to the introduction of EU standardised data collection regulations in 2000 [60].

Various methods have been proposed for improving the expansion from observer data to regional discard estimates—for example, the use of selectivity data to reconstruct discards from landings or as part of stock assessment procedures [61–62], the use of covariates [49] and techniques for identifying outliers among discard samples and improving the precision of regional averages [6]. However, all these approaches still rely on the problematic expansion from landings to catch using observed data on the proportion discarded. It is clear that only limited further information on the past and current extent of overall discarding can be gleaned by such approaches. Moreover, these methods will become unworkable as jurisdictions begin to introduce legal landing obligations on fisheries. As soon as discarding of any species becomes an illegal activity, placing observers aboard fishing vessels to gather data on species which can still be legally discarded will become impossible, since the observer cannot any longer be impartial and the relationship with the vessel skipper will be compromised. For all these reasons, a fresh approach is needed to draw-in additional data [63].

Incorporating additional information on the length-composition of landings ought to provide a basis for a more refined estimate of discards given knowledge of length-dependent retention probabilities. Landed length compositions are monitored at ports typically only for a few major targeted species. Unfortunately these data are often difficult to access since the standard reporting for input to stock assessments is in the form of derived age-compositions. Reconstruction of discard estimates from age-based landings data requires some problematic assumptions about discard rates in relation to age rather than size, or approximate reconstructions of landed length-compositions from age-compositions. In any case, there are no data on length distributions for the majority of landed species; only the total landed weights. Here, we have addressed the problem of estimating discards from a different perspective by developing a statistical model to hind-cast time series of the quantities of catch discarded for individual species, using data on landed weights and length distributions recorded from scientific research vessel trawl surveys. In addition to providing a comprehensive assessment of discarding rates and quantities, incorporating poorly sampled species, the method offers the prospect of estimating discard rates in a future legal framework where deploying observers aboard fishing vessels becomes impractical.

## Overview of Model, Data and Methods

We used the North Sea as a case study region on account of its global significance in terms of discarding by mixed demersal fisheries [5,64]. We develop a model in which the weighted sum of the biomass-at-length distribution from a research vessel survey is assumed to be proportional to the total catch, where the proportionality parameter will be a product of the efficiency of the survey and the harvest rate of the fishery. The weights in this case will be dependent on the selectivity of the commercial fishery and will affect the smallest size classes. We also assume that the discard portion of the catch is the weighted sum of the biomass-at-length where the weights will depend on size selection as the catch is sorted and any bulk discarding of fish, regardless of size. The model therefore requires parameters which describe the selection by the fishing fleet and the post-capture selection by fishers.

To estimate the various selectivity parameters required to calculate the size specific weights, we fitted the model to species for which data on both weight landed and weight discard were available, and used prior estimates of the size below which fish are discarded (the “retention” length). Fitting the model to these “reference” species also provided estimates of survey efficiency. Given estimates of these values, the model was then fitted to species for which only weight landed, the survey size distribution and retention length were available.

We performed various validation checks on the model using independent data. First, we performed a cross validation, in which we predicted the discard quantities of the reference species and compared them with the known discards. Then, for these reference species, we assembled data from stock assessments on the annual proportion of catch for which the motivation for discarding was unrelated to the size of fish, and compared these with the equivalent predictions from the model. Finally, we assembled fragments of data from the literature on the proportion of the catch discarded for as many of the non-reference species as possible, and compared these with the model predictions. At the end of these various data assembly and modelling tasks, we were able to compile a comprehensive summary of the annual quantity and composition of demersal fish catch and discards in the North Sea between 1978 and 2011.

### Discard model

**Conceptual model.** Let us assume that a proportion, *p*, of the total catch, *C*, is discarded. If *D* represents these discards, we may write for a particular year, *t*;

$$D_{t}=p_{t}C_{t}$$ (1)

One may then simply express the landed portion, *L*, as:

$$L_{t}=\left(1-p_{t}\right)C_{t}$$ (2)

In general, only *L* is known and in order to estimate *D* we need to know either *C* or *p*. Using an estimate of *p* to calculate discards from the landings, however, can result in large errors because as *p* nears its upper bound, (1—*p*) approaches zero and we can see from equation (2) that this results in a very large inflation factor which will magnify any small errors in the estimate of *p*. Consequently we focus here on estimating the total catch directly and then calculate the discards by subtracting the known landings.

Discarding will depend on a number of factors that influence the vessel‘s choice to retain fish and will be related, *inter alia*, both to fishery regulations and economic incentives. We consider two main processes that result in discarding. Firstly, fish may be discarded according to *size* because they are too small to be landed either for regulatory or market reasons. Secondly, they may be discarded according to *quantity* because the vessel has exhausted its quota for that species, or because the fish have little market value and space aboard the vessel is limited. Hence total discards will be the sum of the size and quantity related processes.

Since some of the discarding process is related to size it is necessary to consider the size composition of the catch. The total annual catch in weight, *C*, from a particular stock might be represented as the sum of the catches, *c*_*i*_, in each size class, *i*, that are retained by the fishing gear over the size range *l*_min_ to *l*_max_.

$$C_{t}=\sum_{l_{\text{min}}}^{l_{\text{max}}}c_{i,t}$$ (3)

If *B* is the total biomass of fish encountered by the fishing fleet during the year, π_i_ is the proportion of the biomass in each size class, *s*_*i*_ the proportion of these fish retained by the fishing gear (which we refer to as “capture selectivity”), and F_t_ is the harvest rate due to fishing, then the catch in each size class *c*_*i*_ may be approximated by:

$$c_{i,t}=F_{t}\;B_{t}\;s_{i}\;\pi_{i,t}$$ (4)

and hence

$$C_{t}=F_{t}B_{t}\sum_{l_{\text{min}}}^{l_{\text{max}}}s_{i}\pi_{i,t}$$ (5)

The catch in each size class is processed by the fishers to select fish which will be retained for landing and those which will be discarded. We assume that a proportion, *q*, of fish in all size classes is discarded as a result of quantity, regardless of size. In addition, there is a size-related proportion, (1—*r*_*i*_), that is also discarded, where *r*_*i*_ is a function of size and which we refer to as “retention selectivity”. Then the biomass of fish discarded, *d*_*i*_, in each size class is:

$$d_{i,t}=q_{t}c_{i,t}+\left(1-q_{t}\right)\left(1-r_{i,t}\right)c_{i,t}$$ (6)

Substituting (4) into (6) and summing over size classes leads to:

$$D_{t}=F_{t}B_{t}\left[\sum_{l_{\text{min}}}^{l_{\text{max}}}s_{i}\pi_{i,t}+\left(q_{t}-1\right)\sum_{l_{\text{min}}}^{l_{\text{max}}}r_{i}s_{i}\pi_{i,t}\right]$$ (7)

**Statistical model.** In reality, the biomass density distribution of the stock of fish in the sea is unknown and unobservable. However, surveys involving sampling the population by standardised trawl tows provide an index of the stock biomass density.

Suppose we have an abundance index, *u*, obtained from a research vessel survey, that is proportional to the mean biomass in the sea such that:

$$B_{t}=Au_{t}$$ (8)

where *A* is the proportionality constant. Substituting (8) into (5) and defining a catch ratio *Q*_*t*_ *= AF*_*t*_

we have:

$$C_{t}=u_{t}Q_{t}\sum_{l_{\text{min}}}^{l_{\text{max}}}s_{i}^{\prime }\pi_{i,t}$$ (9)

and

$$D_{t}=u_{t}Q_{t}\left(\sum_{l_{\text{min}}}^{l_{\text{max}}}s_{i}^{\prime }\pi_{i,t}+\left(q_{t}-1\right)\sum_{l_{\text{min}}}^{l_{\text{max}}}r_{i}s_{i}^{\prime }\pi_{i,t}\right)$$ (10)

Here, *s'* represents the length selectivity of the fishing fleet relative to the biomass distribution as perceived by the survey, rather than as relative to the biomass distribution in the sea (as in eq. 4 and 5).

For convenience, we assume that *Q* and the biomass index, follow a random walk with a multiplicative error:

$$Q_{t}=Q_{t-1}\;\text{exp}\left(e_{Q,t}\right),\;e_{Q,t}∼N(0,\sigma_{Q})$$ (11)

$$u_{t}=u_{t-1}\;\text{exp}\left(e_{u,t}\right)\;,\;e_{u,t}∼N(0,\sigma_{u}$$ (12)

Here, *σ*_*Q*,_ and *σ*_*u*_ are the standard deviations of the process error which we represent by a normal distribution (*N*). This places some constraints of the estimates of *Q* and *u* and separates process noise from measurement error.

Equation (10) shows that it is possible to calculate the discards from a survey index of the distribution of biomass by size, if the survey catch ratio (*Q*), capture and retention selectivities (*s'* and *r*), and the level of quantity-related non-selective discarding (*q*), are all known. Hence, to complete the model specification, we assume conventional selectivity ogives for *s'* and *r* based on a logistic function parameterised in terms of a 50% selection length and a selection interval (*s'* in terms of *SL*_50_ and *SI*; *r* in terms of *RL*_50_ and *RI* respectively) [65,66]. The choice of a logistic form here is assumed because the majority (92%) of North Sea demersal fish landings are caught with towed gears (otter trawl, beam trawl and seine) [67] that typically show logistic selection characteristics. For the capture process we define selectivity in linearised form as:

$$\text{ln}\left(\frac{s_{i}^{\prime }}{1-s_{i}^{\prime }}\right)=\left(\frac{\text{ln}\left(9\right)}{SI}\right)l_{t}-\frac{SL_{50}\left(\text{ln}\left(9\right)\right)}{SI}$$ (13)

and for the retention selectivity ogive we have:

$$\text{ln}\left(\frac{r_{i}}{1-r_{i}}\right)=\left(\frac{\text{ln}\left(9\right)}{RI}\right)l_{t}-\frac{RL_{50}\left(\text{ln}\left(9\right)\right)}{RI}$$ (14)

The quantity *q* will be driven by quota restrictions and the economic value of the species. These change from year to year and hence will be time dependent and hard to predict. Hence, *q* is treated as an annual random effect.

Finally, it is possible to partition the discards into those selected on the basis of size, and those due to quantity discarding using:

$$\text{Quantity selected component}\;=\;q_{t}C_{t}$$ (15)

$$\text{Size selected component}\;=D_{t}-q_{t}C_{t}$$ (16)

### Parameter estimation

**Fig 1:**
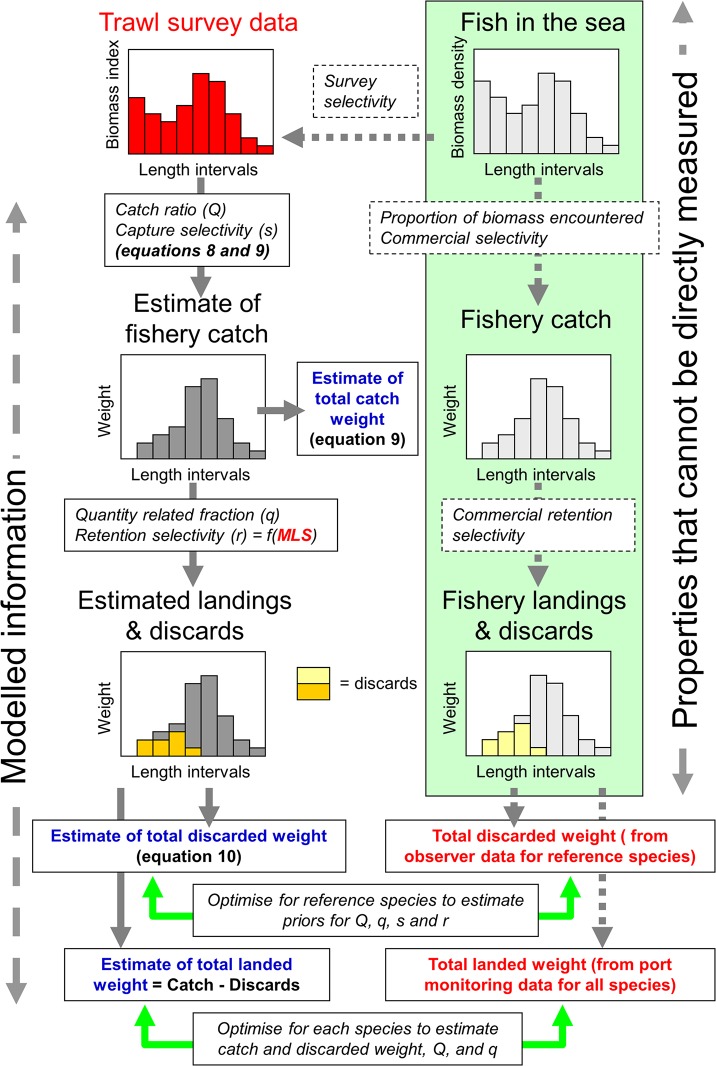
Conceptual basis of the statistical model. On the right, the abundance and length distributions of fish in the sea, the distribution of the fishery catch, and the partitioning of lengths between landings and discards, are essentially unobserved. All we know is the biomass of fish which are landed—and for the reference species the biomass discarded. On the left, we have information on the abundance and lengths of fish in the trawl surveys. From this we can estimate the biomass and length composition of catch and the partitioning between landings and discards, given information on the catch ratio, capture and retention selectivities. Equation numbers refer to equations in the text.

**Full model for species where both landings and discard data are available.** The data typically available for most stocks comprise observed landings, *L'* and an observed survey index *u'*. These data alone are insufficient to estimate *Q*, *s'* and *r* adequately so the approach is to use data for the five main species targeted by the fisheries, which we refer to as ‘reference species’ (cod, haddock, whiting, plaice and sole), for which both landings and discard data exist. The availability of discard data for these species allows us to estimate *Q* and the capture and retention selectivity parameters, which can then be applied to other species (Fig. 1; Table 1).

**Table 1:** Estimated parameters in the full model fitted simultaneously to the five reference species for which both landings and discards data are available, and the choice of priors.

**Parameter**	**Description**	**Prior**	**Basis**
SL_50,k_	50% selection length for capture of species k	Uniform(5,30)	5cm is typically the lowest size class in the data. The upper limit lies well above the size of full retention for most species.
σ_SL50,k_	Standard deviation of random effect for SL_50_ on species k	Uniform(0,100)	Uniform prior over plausible values
SI_k_	Selection interval for capture of species k	Normal(6,1.414)	From [20]. Applies to all reference species.
RL_50,k_	50% selection length for retention of species k	Normal(MLS_k_, 7.76)	From [20]. MLS_k_ is the minimum landing size for species k.
RI_k_	Selection interval for retention of species k	Normal(6,1.414)	From [20]
q_t_	Quantity discard factor	Beta(0.5,3)	Prior has a mode at zero and has a long right hand tail. Assumes low probability of “over quota” discarding.
1/(σ_l,k_)^2^	Precision of landings data	Gamma(0.1,0.1)	Non-informative prior
1/(σ_d,k_)^2^	Precision of discard data	Gamma(0.1,0.1)	Non-informative prior
1/(σ_b,k_)^2^	Precision of survey index data	Gamma(0.1,0.1)	Non-informative prior
Log(u_1,k_)	Log of biomass index in initial year for species k	Normal(u_k_*,0.5)	u_k_* is the mean of the observed biomass index for species k
Log(Q_1,k_)	Log of catchability ratio in initial year for species k	Uniform(2,14)	Non-informative prior
1/(σ_Q,k_)^2^	Precision of survey catchability ratio random walk process	Gamma(0.1,0.1)	Non-informative prior
1/(σ_u,k_)^2^	Precision of survey index random walk process	Gamma(0.1,0.1)	Non-informative prior

For the five reference species, *k*, we define observation equations for the time series of survey indices, landings and discards, *D′*, as:

$$u\prime_{k,t}=u_{k,t}\;\text{exp}\left(e_{b,k,t}\right)\;,\;e_{b,k,t}∼N(0,\sigma_{b,k})$$ (17)

$$L\prime_{k,t}=L_{k,t}\;\text{exp}\left(e_{l,k,t}\right)\;,\;e_{l,k,t}∼N(0,\sigma_{l,k})$$ (18)

$$D\prime_{k,t}=D_{k,t}\;\text{exp}\left(e_{d,k,t}\right)\;,\;e_{d,k,t}∼N(0,\sigma_{d,k})$$ (19)

where the term *e* represents the measurement errors associated with the observed data.

Given observed values, *L′* and *D′* and a survey index *u′*, it is now possible to estimate the capture and retention selectivity parameters, survey catch ratio and non-selective discard fraction, using the likelihoods resulting from (eq. 17–19). One approach is to apply the model separately to each of the five species to obtain species specific selectivity parameters. However, since we wish to apply these values to other species it is useful to consider “pooled” values for the commercial capture selectivity to obtain a “typical” set of selectivity parameters that could be more widely applied. We assume therefore that for each species we can describe the length at 50% capture efficiency as the sum of a common mean (*SL* ^*^_50_) plus a species specific random effect. Hence for species *k* we have:

$$SL_{50k}=SL_{50}^{*}+e_{SL50k}\;,\;\text{where}\;e_{SL50,k}∼N(0,\sigma_{SL\mathrm{50}})$$ (20)

**Reduced model for species where no discard data are available.** Where only the biomass index and landings data are available it is not possible to obtain reliable estimates for capture or retention selectivity parameters. In order to estimate discards it is necessary to fit the model with informative priors on the capture parameters and to specify the 50% retention length (Fig. 1; Table 2). We used the posterior distribution for *SL* ^*^_50_ obtained from the reference case and applied this to all other species as the approximate fleet selectivity parameter. Similarly for *Q* we used the estimated values *Q*_*k*,*t*_ drawn from the posterior distributions for reference species *k* in year *t* from the reference case to calculate a weighted average. We assumed a geometric mean, given a skewed distribution of *Q*-values for the reference species:

$$\text{log}\left(Q_{t}\right)=\sum_{k}w_{k}\;\text{log}\left(Q_{k,y}\right)$$ (21)

Here, the weights, *w*_*k*_ are constrained by $\sum_{k}w_{k}=1$ and are treated as parameters to be estimated.

**Table 2:** Estimated parameters in the reduced model for a single species where only landings data are available, and the choice of priors.

**Parameter**	**Description**	**Prior**	**Basis**
SL_50_	50% selection length for capture	Normal(15.25,4.93)	Posterior from reference case
SI	Selection interval for capture	Normal(6,1.414)	From [20]
RI	Selection interval for retention	Normal(6,1.414)	From [20]
q_t_	Quantity discard factor	Beta(1,1) or Beta(1.4,12)	Uniform prior for low value species, or posterior distribution of q from the reference case for high value species.
1/(σ_l_)^2^	Precision of landings data	Gamma(0.1,0.1)	Non-informative prior
1/(σ_b_)^2^	Precision of survey index data	Gamma(0.1,0.1)	Non-informative prior
1/(σ_u_)^2^	Precision of survey index random walk process	Gamma(0.1,0.1)	Non-informative prior
Log(u_1_)	Log of biomass index in initial year	Normal(u*,0.5)	u* is the mean of the observed biomass index
w_k_	Weight given log(Q_k_)	Dirichlet(1,1,1,1,1),	Non-informative prior

The 50% retention length for landing was taken to be proportional to the species legal or *de-facto* minimum landing size (MLS) where the proportionality constant, *h*, was calculated as the ratio of the mean *RL*_50_ to the mean MLS for the five species estimated from the reference case, i.e.

$$RL_{50}=h(MLS),\;\text{where}\;h=\frac{\sum_{k}RL_{50,k}}{\sum_{k}MLS_{k}}$$ (22)

For some species there may be years where the observed survey biomass index is zero but there are recorded landings. Zero indices were therefore treated as missing values. However, this leads to problems in estimating discards when there is no size composition available. For these years an average size composition was used and the *q* value used was the mean of the prior distribution on *q*.

### Model input data

**Scientific trawl survey data.** Data to form an index of fish abundance at length (u_i,t_) were assembled from the North Sea International Bottom Trawl Survey (IBTS) [68] conducted during quarter 1 (January—March; Q1) each year. The data, which were downloaded from the ICES DATRAS data centre [69] included records collected with a variety of survey gears since 1965. However, for consistency of species and size selectivity, we confined our analysis to the data collected between 1978 and 2011 and within the geographic region 4°W—10°E and 51°N—62°N (S1 Fig.). The methods and spatial coverage have been internationally standardised using a Grande Overture Vertical (GOV) otter trawl during this period. In each survey, all or a random subsample of the fish of each species caught in the trawl net are counted and measured to at least the nearest cm-below. In total, the selected data from the IBTS comprised 1.179 million records of species/length class abundances from 12,323 tows.

**Official landings data.** Official statistics on the total annual landed weights of all species, by all nations engaged in fisheries in North Sea regions IVa, IVb and IVc combined, between 1978 and 2010 were accessed from the FAO/ICES FishSTAT dataset [70]. National statistics were combined to estimate the total international landed weight. In some cases species were aggregated into broader taxonomic groups to accommodate national and temporal differences in the way species were recorded, such as general categories for “sharks” or “rays” (S1 Table)

**Legal minimum landing sizes.** Data on legal minimum landing sizes (MLS) were compiled from EU fisheries legislation documents and national information issued to the fishing industries. Internationally recognised minimum legal landing sizes of some fish species exploited in the North Sea were instituted in 1983. Limits have since been revised from time-to-time and additional species included in the legislation (Table 3). For cod, haddock and plaice, minimum legal landing sizes formed two distinct eras, pre- and post-1989 when an increase was legislated for all three species (cod: 30 and 35cm (pre- and post-1989); haddock: 27 and 30cm; plaice: 25 and 27cm). For whiting, the corresponding eras were pre- and post-1979 (25 and 27cm).

**Table 3:** Legal minimum landing sizes of North Sea fish species under UK Statutory Instruments (SI) and European Council Regulations (CR) post-introduction of the Common Fisheries Policy in 1983.

**Species**	**Legal minimum landing size (cm)**	**Effective date (d/m/y)**	**Legal document reference**
Cod	30	1/4/1961	[102]
Cod	35	1/1/1989	[103]
Haddock	27	1/4/1961	[102]
Haddock	30	1/1/1989	[103]
Whiting	27	1/7/1979	[104]
Whiting	25	1/1/1989	[103]
Whiting	23 (27—the UK maintained a whiting MLS of 27cm as a derogation from an EU Council Regulation that reduced it to 23cm)	1/6/1992	[105,106]
Whiting	27	1/1/2000	[107]
Plaice	25	1/4/1961	[102]
Plaice	27	1/1/1989	[103]
Saithe	30	1/1/1982	[108]
Saithe	35	1/1/1989	[103]
Anglerfish	500 g ≈ 32cm	1/1/1997	[109]
Common sole	24	1/1/2000	[107]
Hake	27	1/1/2000	[107]
Megrim	20	1/1/2000	[107]
Ling	63	1/1/2000	[107]
Blue ling*	70	1/1/2000	[107]
Pollack	30	1/1/2000	[107]
Bass	36	1/1/2000	[107]

SI and CR that merely maintained legal landing sizes at current values are not listed.

*Although blue ling have an assigned legal minimum landing size, they are only rarely caught in the IBTS trawl surveys, so were included in the ‘other marketable’ species category for analysis with the reduced model.

**Effective or *de-facto* minimum landing sizes.** Species which are not covered by legal minimum landing size legislation are nevertheless subject to *de-facto* effective minimum sizes dictated by market demand, which we estimated from fishing industry documents and published data on landed length distributions assembled by the ICES [71]. Failing these sources, we estimated values from anecdotal information (Table 4).

**Table 4:** *De-facto* landing sizes of species for which there is no legal minimum.

**Species**	***De-facto* minimum landing size (cm)**	**Source reference**
Common dab	25	[71,110]
Lemon sole	25	[71,110]
Witch	28	[71]
Brill	30	[71]
Flounder	27	[71]
Gurnard	30	[110]
Mullets	16	[110]
Turbot	30	[71,73,110]
Halibut	45	Anecdotal
Dogfishes and sharks	50	[110]
Spurdog	50	[110]
Rays and skates	40	[110]
Wolffish	30	Anecdotal
Tusk	40	Anecdotal
Other marketable	30	Anecdotal

### Model fitting data

**ICES landed and discarded weights for the reference species.** ICES assessment working groups routinely calculate the total international annual weights landed (*L*_*t*_) and discarded at sea (*D*_*t*_) for the most intensively targeted demersal stocks in the North Sea (cod, haddock, whiting and plaice and sole) from combinations of statistics collected at fishing ports and by observers on vessels at sea. The ICES landings data may differ from the official statistics where local knowledge indicates ‘unallocated landings’, comprising mis-reported or undeclared landings, which were not set against any national landing quotas.

For cod, haddock and plaice, we collated the total international data for the years 1978–2010 given in the 2011 ICES Advice Book [72]. Data for whiting prior to 1989 were unavailable from this source, so we used values from an earlier report [73].

ICES data on landings and discards of sole in the North Sea are less complete than for the other four species. We used landings from 1982–2010 from the 2011 ICES Advice Book [72], supplemented with 1978–1981 data from official statistics. ICES bases discard weights of sole on the proportions of catch discarded by Dutch registered beam trawlers [74]. We used the values quoted by ICES [59] to derive estimates of discarded weights from landings (*D*_*t*_ *= p*_*t*_.*L*_*t*_ */*(1—*p*_*t*_)).

The assembled data on all five species are provided as S2 Table.

### Model validation data

**Catch and discard age compositions for the reference species.** In addition to compiling total landed and discarded weights, ICES working groups also assemble data on annual numbers-at-age and mean individual body weight-at-age for the landed and discarded components of the catches of the main fishery species. These are required as input to the stock assessment models. We extracted these data from the 2012 North Sea Demersal Working Group report [59]. Unfortunately, the original length distribution data which were collected by national sampling schemes in order to compile these internationally aggregated data are not available from ICES.

**Discard weights of other (non-reference) species.** Data on multi-year average, individual year, or anecdotal data on discard rates for 25 species or species groups other than the five main targets were assembled from a wide range of the literature and data reports originating from national monitoring schemes (S3 Table).

**Observer data on length distributions of catch and landings.** Although North Sea wide, internationally compiled length distributions of landings and discards are unavailable even for the reference species, limited data from some national observer programmes are available. Two data reports from the European Commission and ICES [71,75] provided observer data on length frequency distributions of captured, discarded and landed fish for a variety of species, which we used to independently estimate the retention selectivity parameter *RL*_50_. Limited additional data were located in published literature [76].

## Methods

### Processing of model input data

**Aggregation of species data to a common taxonomic resolution.** We first identified the subset of demersal species categories in the IBTS survey data which corresponded with species categories in the ICES/FAO landings data. All other demersal species in the surveys were grouped together as a ‘discard-only’ class, for which we assumed *p* = 1. Of the remainder, a subset of 26 species groups formed from 76 individual IBTS taxa (including the 5 reference species cod, haddock, whiting, plaice and sole) which comprised up to 98% of the cumulative landed weight over the study period 1978–2011, were treated as independent entities. The remainder (≤ 2% of landed weight) were combined as a group formed of 67 IBTS taxa, which we refer to as ‘minor marketable’ species. (S1 Table).

**Estimation of mean number density in survey catches.** The area swept by each tow of a survey gear was given by the product of distance towed and the separation distance of the wing-ends of the GOV trawl. Swept area was derived from the trawl geometry data where available, and imputed from statistical relationships with duration and seabed depth where values were unrecorded (ICES 2010). For each survey year, all hauls located in the ICES North Sea regions IVa, IVb and IVc combined were extracted from the data set, and the annual averages of number density per 1cm length class calculated for each taxonomic class and year. Taxon abundances were collapsed to the highest level of resolution compatible with differences in temporal and national variations in the way species were recorded.

**Conversion of survey number density to biomass density.** The number density in each annual species/length class was converted to biomass density by application of a time-independent length-body weight conversion for each species (*body weight = a*.*length* ^*b*^). The parameters *a* and *b* were compiled from literature sources [77,78] (see S4 Table).

### Processing of model validation data

**Estimation of retention selectivity from observer data.** Observational data on dab and gurnard catch and landings length distributions from ICES WGNEW [71] were sufficient to estimate the proportion of catch landed-at-length. For these data we fitted eq. 14 to estimate *RL*_50_.

Species length distributions from the EU [75] were standardised as proportions cm^−1^ for discards and landings separately, which meant that information on the relative abundances of fish discarded and landed was lost. Hence for these data it was not possible to derive proportion of fish landed in relation to length (*L*_*i*_ */C*_*i*_). However, we roughly estimated selection interval (*RI*) as the difference between the minimum length of landed fish and the maximum length of those discarded, and the 50% retention length (*RL*_50_) as mid-way between these limits.

**Estimation of reference species quantity-related discard fraction (*q*) from observed age compositions of catch and discards.** For each of the four reference species where observed age compositions of catch or landings and discards were available (cod, haddock, whiting, plaice (not sole)), we estimated the discard rate (proportion by weight) of fish larger than a size threshold. The threshold was set at the MLS plus an increment of 1 cm (∼5%) to account for the selection interval. This fraction should be equivalent to the quantity-related discard term *q* in our model.

Using the same length-body weight parameters as in the conversion of survey numbers-at-length to survey weight-at-length (see above), we converted the threshold length to a threshold body weight. For each year of data, the total weights caught and discarded were then summed over all age classes where the mean body weight exceeded the threshold. Clearly, the use of a knife-edge selection threshold in this way gives only an approximate estimate of *q* since it assumes that the entire weight of an age class in the catch or discards is either larger or smaller than the threshold while, in reality, each class has a distribution of sizes. However, in the absence of data we can only guess at these distributions.

### Model fitting

We used OpenBUGS [79,80] to fit the full model to the ICES landed and discarded weights for the reference species using priors on the parameters given in Table 1. We refer to this model fit as the ‘reference case’. We ran the model initially with three chains for various iteration lengths and burn in periods. Good chain mixing was achieved after about 10,000 iterations. The final model run used a single chain of 50,000 iterations and a burn in period of 15,000 iterations.

For the reduced model similar exploratory analysis resulted in fits using a burn in period of 50,000 iterations and a chain length of 15,000 iterations. To approximate an informative prior for *q* we used a beta distribution with the same mean and variance as the distribution of posterior mean *q* values for the species in the reference case. Prior distributions for the other parameters are given in Table 2. OpenBUGS code for the reduced model with an example dataset is given in S1 Appendix.

### Model validation

As a check of the reduced model, a cross validation exercise was performed where the model was fitted to the ICES landings data for each of the five reference species, but without involving the discard data. The estimated discards from the model were then compared to the observed values.

We were only able to validate the reduced model for all the other non-reference species in cases where independent observations of proportion discarded existed. However, the problem was that these data consisted of sporadic point estimates or averages over periods of years, for individual national fleets, and often only for restricted geographical areas. There were no international North Sea-wide aggregations of these data. In fact, this may be impractical in any meaningful way where the data are sparse and highly variable between fleets. Hence we opted for a simple approach of comparing the mean discard rate across all observations for each species, with the mean proportion estimated from the reduced model over the period 2001–2010 (the period over which most observations originated). By this means we aimed to assess the overall consistency between the model and the observations, taken across all the species together. We used a logit transformation to calculate the means in view of the restricted scale of the observations.

### Model sensitivity

We conducted a sensitivity analysis on the reduced model to investigate the effect of the most important parameters and assumptions on estimated discards. Parameters included in the analysis were: the 50% capture length for the fishery (*SL*_*50*_); the effective 50% retention length (*RL*_*50*_; eq. 22), and the assumed priors for the values of bulk discarding (*q*). Each of these terms was varied in turn by 10% to determine the effect on estimated discard quantities of each species. An addition, we tested the sensitivity of discard estimates to the assumption of a weighted geometric mean, as opposed to an arithmetic mean, of the reference species values of catch raising factor (*Q*) in eq. 21.

## Results

### Species biomass density in trawl surveys

The biomass densities (kg km^−2^ cm^−1^) of the reference species (cod, haddock, whiting, plaice and sole) captured in the trawl surveys (S2 Fig.) showed that in each case a substantial proportion of the biomass was composed of fish larger than the relevant MLS. However, this was not true for all of the other non-reference species (S3 Fig.). In particular, the most abundant (in terms of biomass) size classes of gurnards, common dab, and minor marketable species were smaller than the corresponding MLS.

### Comparison of estimated 50% retention length with observed values and legal minimum landing sizes

**Fig 2:**
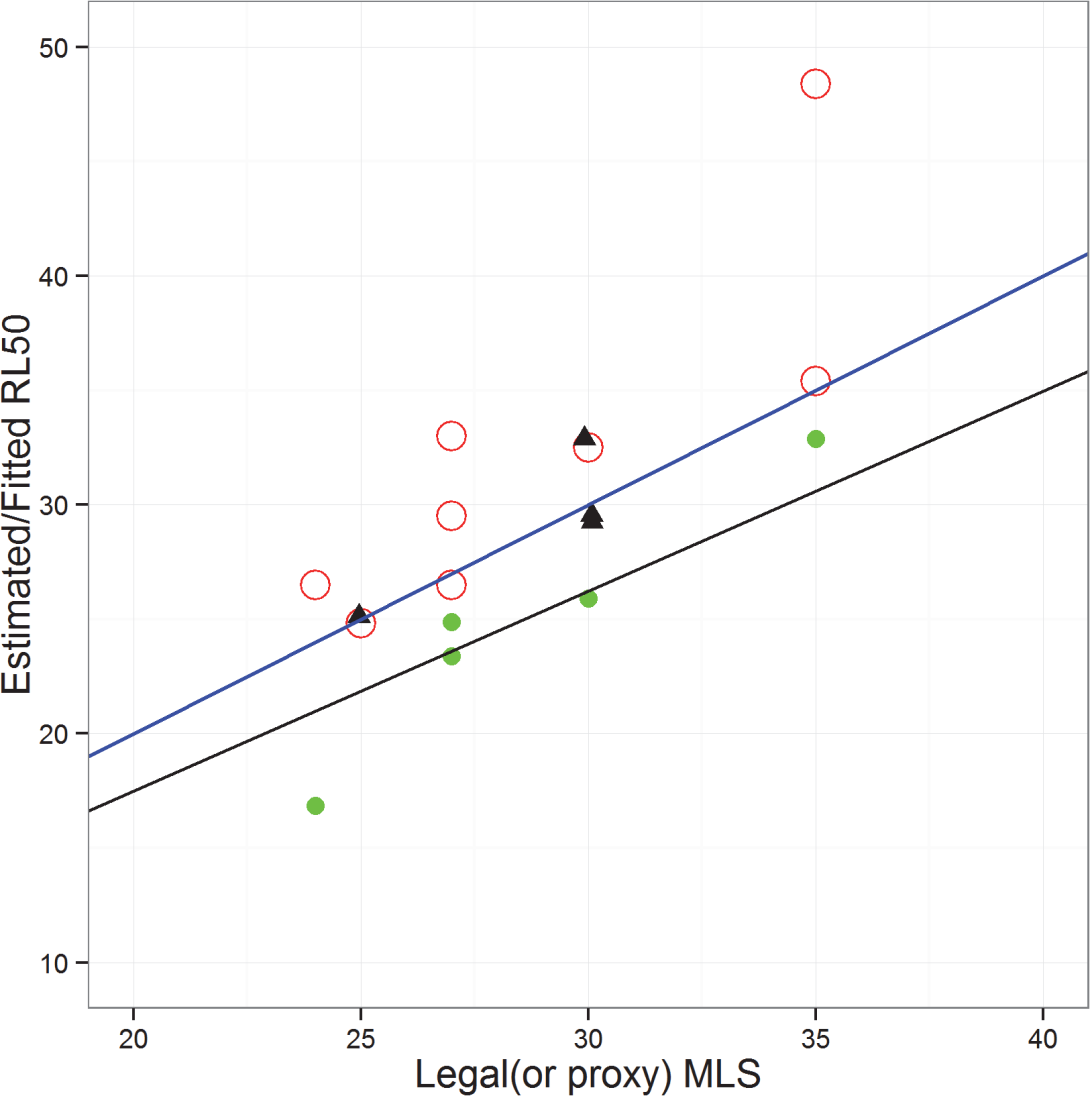
Relationships between legal or *de-facto* minimum landing size (MLS), and estimated or fitted values of *RL*_*50*_ (the length at 50% retention). Red circles: MLS vs observer data estimates of *RL*_*50*_ for species subject to a legal landing size limit (cod, haddock, whiting, plaice, sole, saithe, hake—see S5 Table). Black triangles: *de-facto* MLS vs observer data based *RL*_*50*_ for species with no legal landing size limit (gurnard, dab, turbot, brill. Green symbols: legal MLS vs *RL*_*50*_ for reference species estimated by the full model. Blue line: 1:1 relationship; black line fitted regression through the origin for full model reference species data. Regression coefficient = 0.874 (r^2^ = 0.817).

Legal MLSs for cod, haddock, whiting, plaice, sole, saithe and hake (Table 3), were significantly correlated with mean 50% retention lengths (*RL*_*50*_) derived from observer data (S5 Table) (Fig. 2; r^2^ = 0.984, f-statistic: 374.8 on 1 and 6 d.f., p-value: 1.23 x 10^–6^). The coefficient of a linear regression fitted through these data was not significantly different from 1 (p<0.05), and the intercept was not significantly different from 0 (p<0.05). Hence, for the reference species, we conclude that there was a 1:1 relationship between MLS and the observer-based estimates of 50% retention lengths.

For species with no legal landing sizes (dab, turbot, brill gurnards; Table 4), our estimates of *de-facto* MLS were also significantly correlated with observer-based estimates of *RL*_*50*_ (Fig. 2; r^2^ = 0.999, f-statistic: 2.122 x 10^4^ on 1 and 3 d.f., p-value: 7.133 x 10^–7^). Here also, linear regression showed that the relationship was not significantly different from 1:1 (p<0.05).

For the reference species, 50% retention lengths (*RL*_50_) estimated by fitting the full model were positively related to the corresponding legal MLS (Fig. 2). The parameter *h* (eq. 22) was estimated to be 0.874 (95% confidence interval 0.768–0.980), indicating that *RL*_50_ values estimated by the model were smaller than the MLS. The likely reason for the discrepancy between observed and modelled *RL*_50_ values was that the biomass index in the model represents the size composition in February each year, while the actual catch is taken throughout the year—during which fish are growing. This means that fish below the MLS at the time of the survey can be legally landed later in the year when they have grown. Nevertheless, we conclude that we are justified in estimating 50% retention selectivity for each species on the basis of MLS values using the *h*-parameter (eq. 22).

### Full model fit for the reference species

Assuming time-independent capture and retention selectivity parameters (Table 1) the full model estimated values of the catch ratio parameter *Q* which differed substantially between reference species. Ratios were highest for sole, and lowest for haddock and whiting (S4 Fig.). In all cases there was a declining trend in *Q* over time, reflecting the reduction in fishing mortality affecting these species.

**Fig 3:**
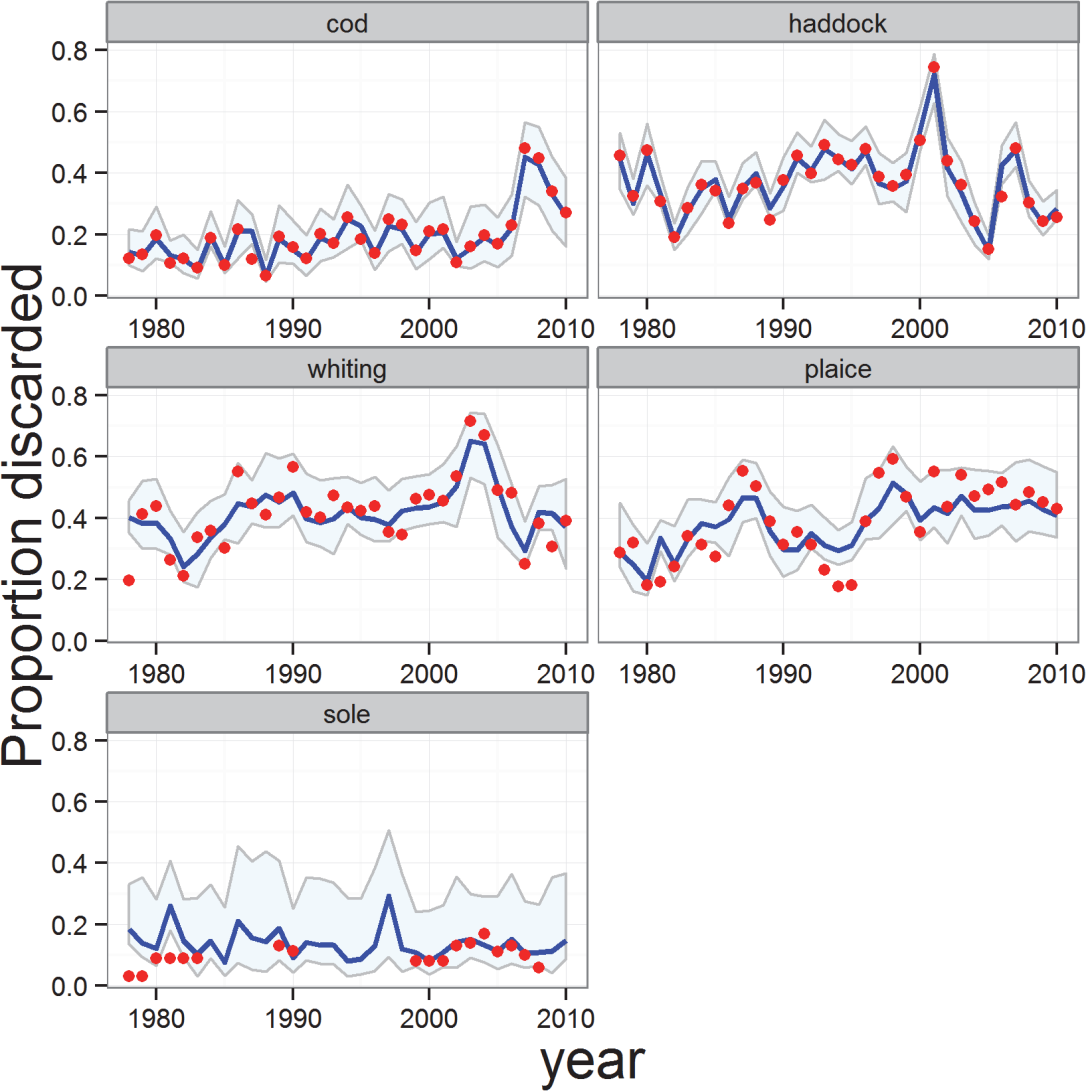
Full model fitted estimates of the total proportions of catch discarded (*p*) for the reference species. Blue line and shading: fitted proportions and 95% credible intervals. Red symbols: observed proportions from ICES stock assessment reports.

**Fig 4:**
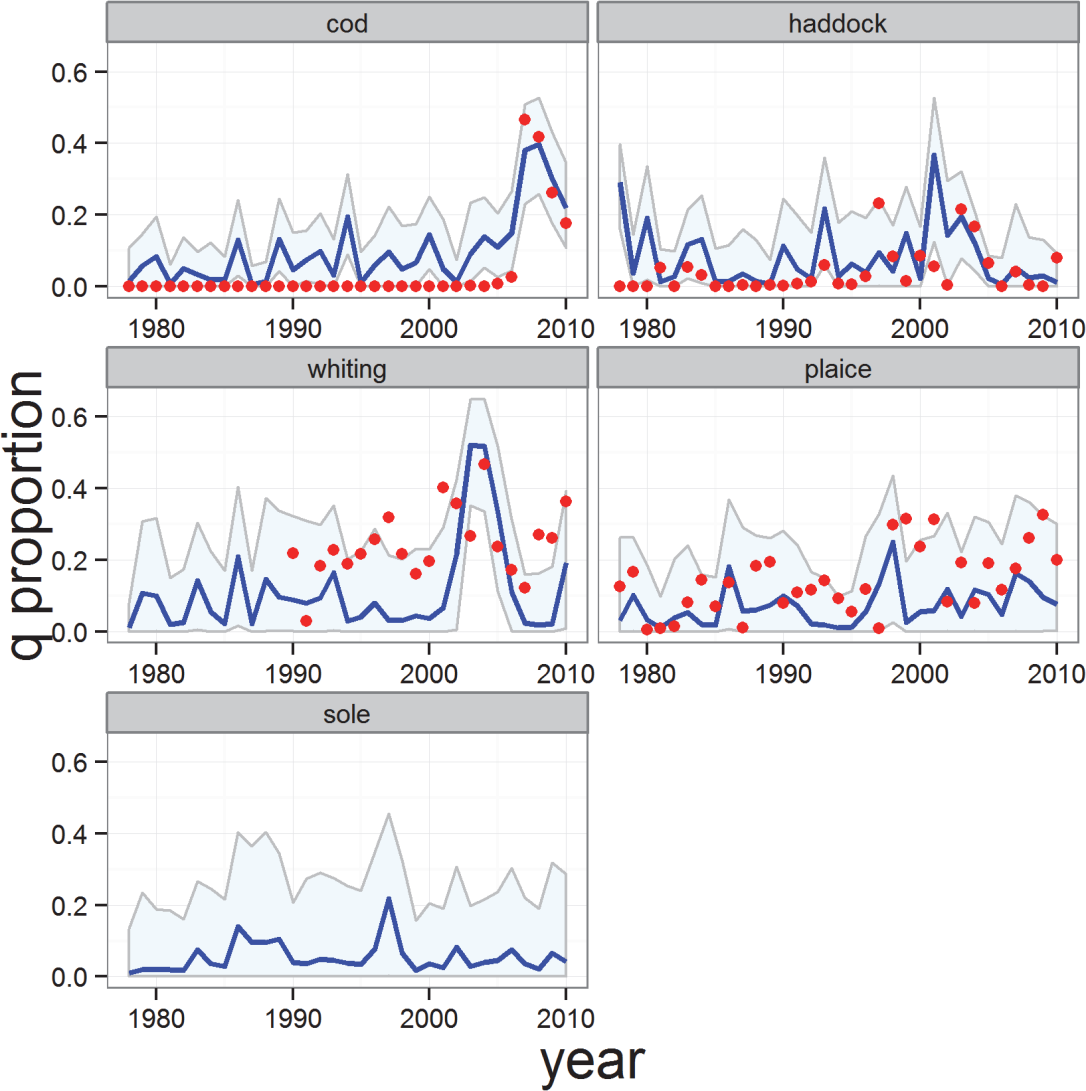
Full model estimates of the quantity-related proportions of catch discarded (*q*). Blue line and shading: median of estimated proportions and 95% credible intervals. Red symbols: quantity-related discard proportions derived from data on catch and discard numbers and mean weights-at-age. In this case, the model was not fitted to the observed data so coincidence between the model and data is an independent validation of the model.

The models provided high-quality fits to the observed data on total proportions of catch discarded for each reference species, with the majority of observed data being contained within the 95% credible intervals for each species. The poorest fit was for sole (Fig. 3). Averaged across all 5 reference species, the median of the fitted model explained 57% of the time series variation in the observed discard weights (79% excluding sole, S6 Table). In addition, the quantity-related proportions of catch discarded (*q*) for each species predicted by the model were consistent with the crudely estimated proportions derived from the ‘observed’ numbers and body weights-at-age in landings and discards (Fig. 4). The results indicated that the majority of the discarding of reference species was size-related and comprised fish smaller than the retention length. This was corroborated by the stock assessment data on age compositions of landings and discards—the weighted mean age of discards was systematically younger than that of the landed component of the catch for each of the main reference species (Table 5). Nevertheless there were periods when quantity-related discarding assumed an important role. In particular, the modelled time series of *q* showed peak values for cod during the late 2000’s, and between 2003–2005 for whiting and haddock (Fig. 4). Modelled values of *q* were almost entirely <0.2 for plaice indicating that quantity-related discarding has rarely been a major issue for this species (Fig. 4).

**Table 5:** Mean ages of cod, haddock, whiting and plaice in discards and landings (weighted by the mass in each age class), averaged over decades.

		**1980–1989**	**1990–1999**	**2000–2009**
**Cod**	**Discards**	1.25	1.46	1.79
	**Landings**	3.13	3.24	3.54
**Haddock**	**Discards**	1.96	1.96	2.52
	**Landings**	3.12	3.22	4.10
**Whiting**	**Discards**	-	2.31	2.50
	**Landings**	-	3.48	3.66
**Plaice**	**Discards**	1.96	2.30	2.34
	**Landings**	4.44	4.75	4.51

Data derived from records compiled for stock assessment [59] No data available for whiting during 1980–1989.

### Cross-validation of the reduced model by application to the reference species

We tested the reduced model by applying it to the five reference species without involving the observed data on discards. The success at recovering the actual discard quantities was then a direct measure of the performance of the reduced model.

**Fig 5:**
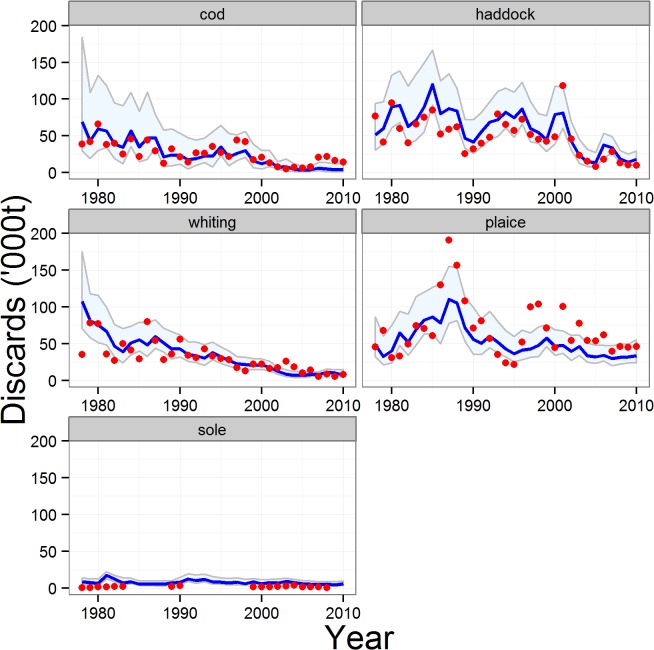
Cross-validation test of the reduced model: discarded weights of the reference species. Blue line and shading: median of estimated weight discarded and 95% credible intervals from the reduced model. Red symbols: observed weights discarded from ICES stock assessment reports. In this case, the model was not fitted to the observed data so coincidence between the model and data is a validation of the reduced model.

The observed discards were almost entirely contained within the credible interval of discard quantities predicted by the reduced model for all reference species except sole (Fig. 5). Coefficients of determination showed that the median of the model outputs for each species explained up to 56% of the time-series variation in the observed discard quantity (S6 Table). In each case the model gave highest weighting to the appropriate reference species for calculating the catch ratio parameter *Q* (Table 6; S5 Fig.), indicating that the landings data contain useful information on abundance, though in the case of cod there was some spread in the weightings.

**Table 6:** Recovery of the appropriate catch ratio (*Q*) in the cross-validation runs of the reduced model.

		***Q*-series from full model runs**				
		**Cod**	**Haddock**	**Whiting**	**Plaice**	**Sole**
**Cross-validation run species**	**Cod**	0.541	0.180	0.124	0.111	0.043
	**Haddock**	0.083	0.596	0.272	0.031	0.018
	**Whiting**	0.060	0.238	0.655	0.029	0.018
	**Plaice**	0.120	0.097	0.087	0.450	0.246
	**Sole**	0.020	0.018	0.017	0.034	0.910

The reduced model estimated weightings to be applied to the reference species *Q*-values from the full model, in order to derive a time series of *Q*-values for each of the other non-reference species. In the cross-validation runs, where we apply the reduced model to the reference species without using the observed data on discards, we expect the model to assign a weighting of 1 to the corresponding reference species and 0 to all the others.

The reduced model consistently over-predicted discards of sole. There were two main reasons for this. First the high catch ratio (*Q*) for sole indicated that this species was under-sampled by the survey relative to the other species. The magnitude of the catch ratio introduced uncertainty into the estimation by scaling up small errors in the survey index. Secondly, the 50% discard retention length estimated from the minimum landing size regression (eq. 22; Fig. 2) was higher than that estimated from the full model indicating that more fish from the catch size composition were below the retention size threshold, and this is the main reason for the increased estimates of discarded fish.

### Application of the reduced model to other species

**Fig 6:**
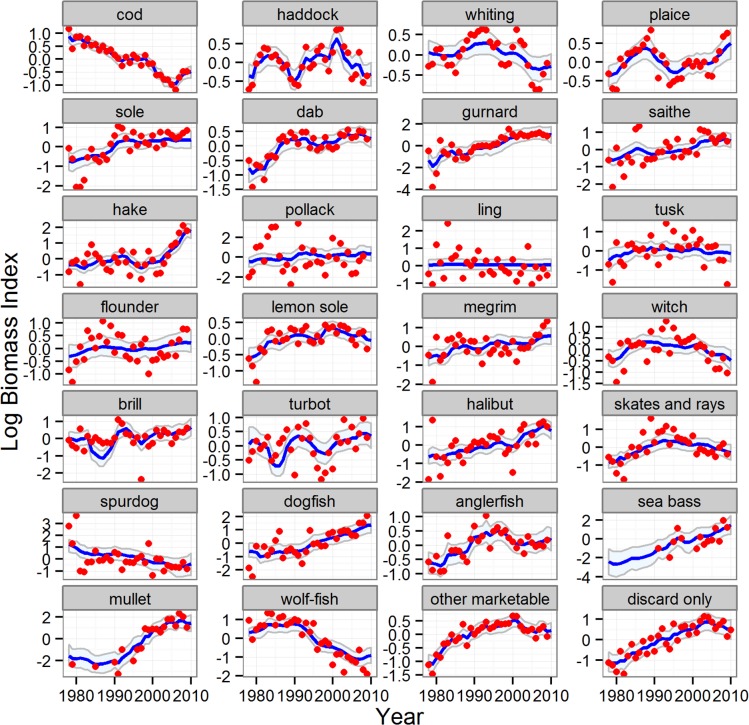
Modelled and observed time series of the survey biomass index (*u*_*t*_) for the reference and other species. Blue line and shading: median of estimated index and 95% credible intervals. Red symbols: observed index. Results for the five reference species from the full model; results for all other species from the reduced model.

**Fig 7:**
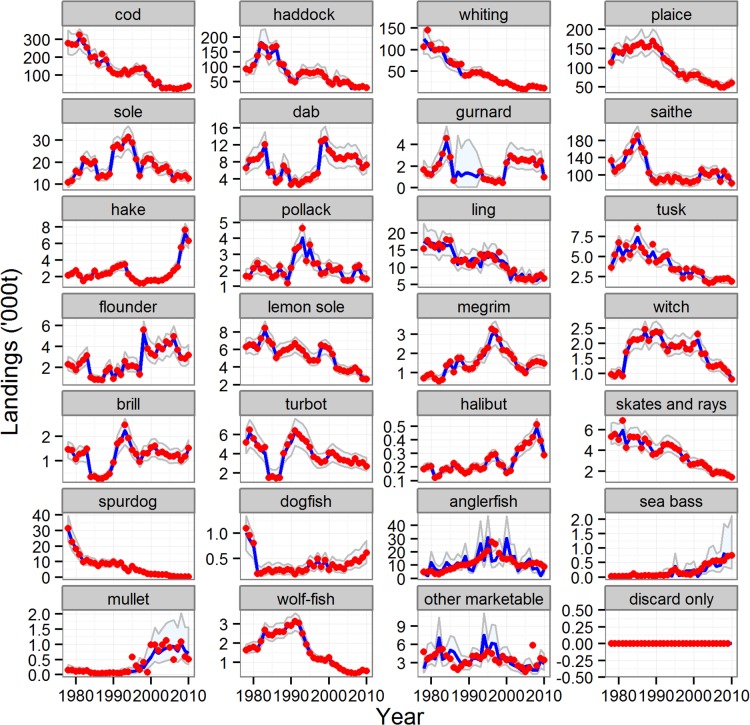
Modelled and observed time series of the weight landed for the reference and other species. Blue line and shading: median of estimated landings and 95% credible intervals. Red symbols: observed weight landed. Results for the five reference species from the full model; results for all other species from the reduced model.

**Fig 8:**
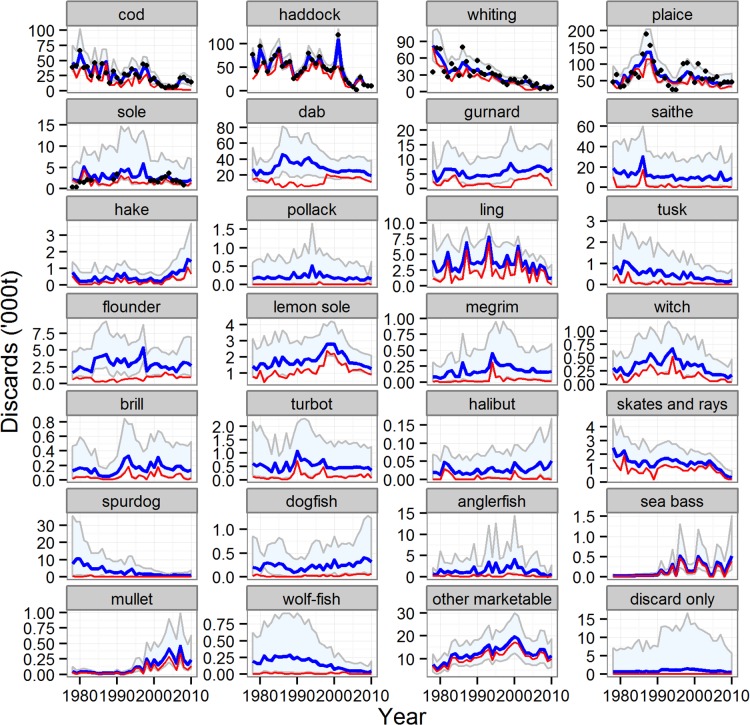
Modelled time series of the weight discarded for the reference and other species. Blue line and shading: median of estimated total discarded weight and 95% credible intervals; red line estimated size-related weight discarded. Black symbols for reference species only: observed weight discarded. Results for the five reference species from the full model; results for all other species from the reduced model.

**Fig 9:**
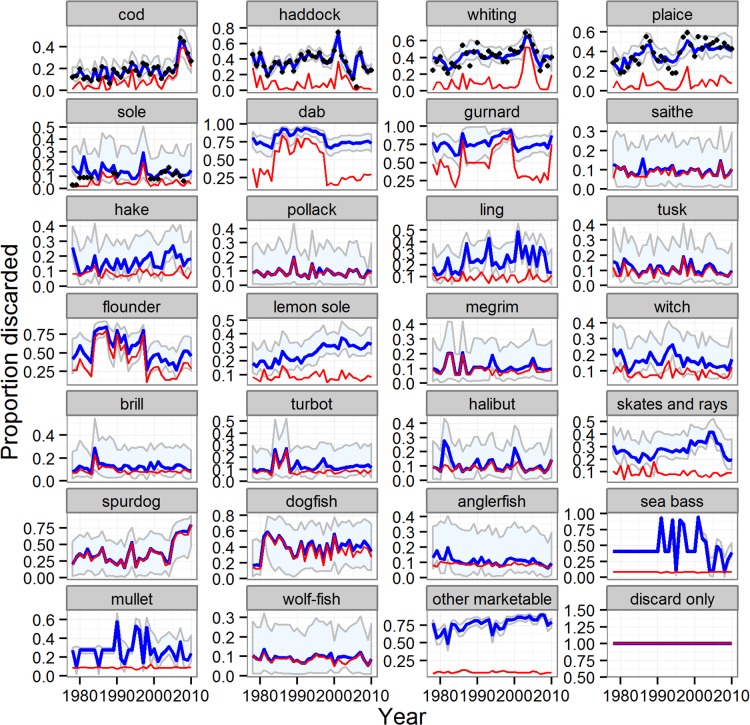
Modelled time series of the proportion of catch discarded for the reference and other species. Blue line and shading: median of estimated total proportion discarded and 95% credible intervals; red line estimated quantity-related proportion discarded. Black symbols for reference species only: observed total proportion discarded. Results for the five reference species from the full model; results for all other species from the reduced model.

There were marked temporal trends in the biomass indices (Fig. 6) and landings (Fig. 7) of the other (non-reference) species, with some clearly increasing and others decreasing, reflecting a progressive shift in the species composition of the stocks in the sea. The reduced model was an extremely effective fit to both the landings time series (average 87% of time series variation explained across all species) and the biomass index (average 49% of time series variation explained across all species, S6 Table). Similarly, discard quantities of many key species (cod, haddock, whiting, plaice, tusk, skates and rays, spurdog) were estimated by the reduced model as decreasing over time, whilst others increased (hake, mullet and bass) (Fig. 8). Most species displayed little or no long-term trend in the proportions of catch discarded, with the exception of cod, plaice, spurdog, hake and lemon sole (Fig. 9). Common dab and gurnards stood out as having high overall discard rates (>0.6), with a high and variable component of quantity-related discarding (Fig. 9).

The estimated weightings given to the reference species catch ratio (*Q*) for each of the non-reference species (eq 21) were partly diagnostic of the quality of the sampling efficiency of the survey. Species which are inefficiently sampled by the survey (e.g. anglerfish, bass, wolffish) received high weightings for sole and plaice (S5 Fig.), whilst the more efficiently sampled species were more heavily weighted to other reference species. For species where the landings data contain little information on abundance, more or less equal weight was given to all reference species. This in effect means that a geometric mean catch ratio was assigned to these species.

### Sensitivity analysis of the reduced model

The estimated discards were only weakly sensitive to assumptions about the capture selectivity parameter (*SL*_*50*_) and the quantity related discard fraction (*q*), showing <5% change on average when these parameters were disturbed by 10%. Similarly, the results were only weakly sensitive to replacement of the geometric mean with an arithmetic mean in eq. 21 for the estimation of catch raising ratios (*Q*). However, the model was strongly sensitive to assumptions about the 50% retention length (RL_50_). A 10% variation in this parameter produced up to 80% variation in discard estimates for species with a small maximum length and narrow length range such as dab, though much smaller variations (∼10%) for species with larger maximum size and wider length range such as cod or saithe.

### Comparison of the modelled discard rates (*p*) with observed data

**Fig 10:**
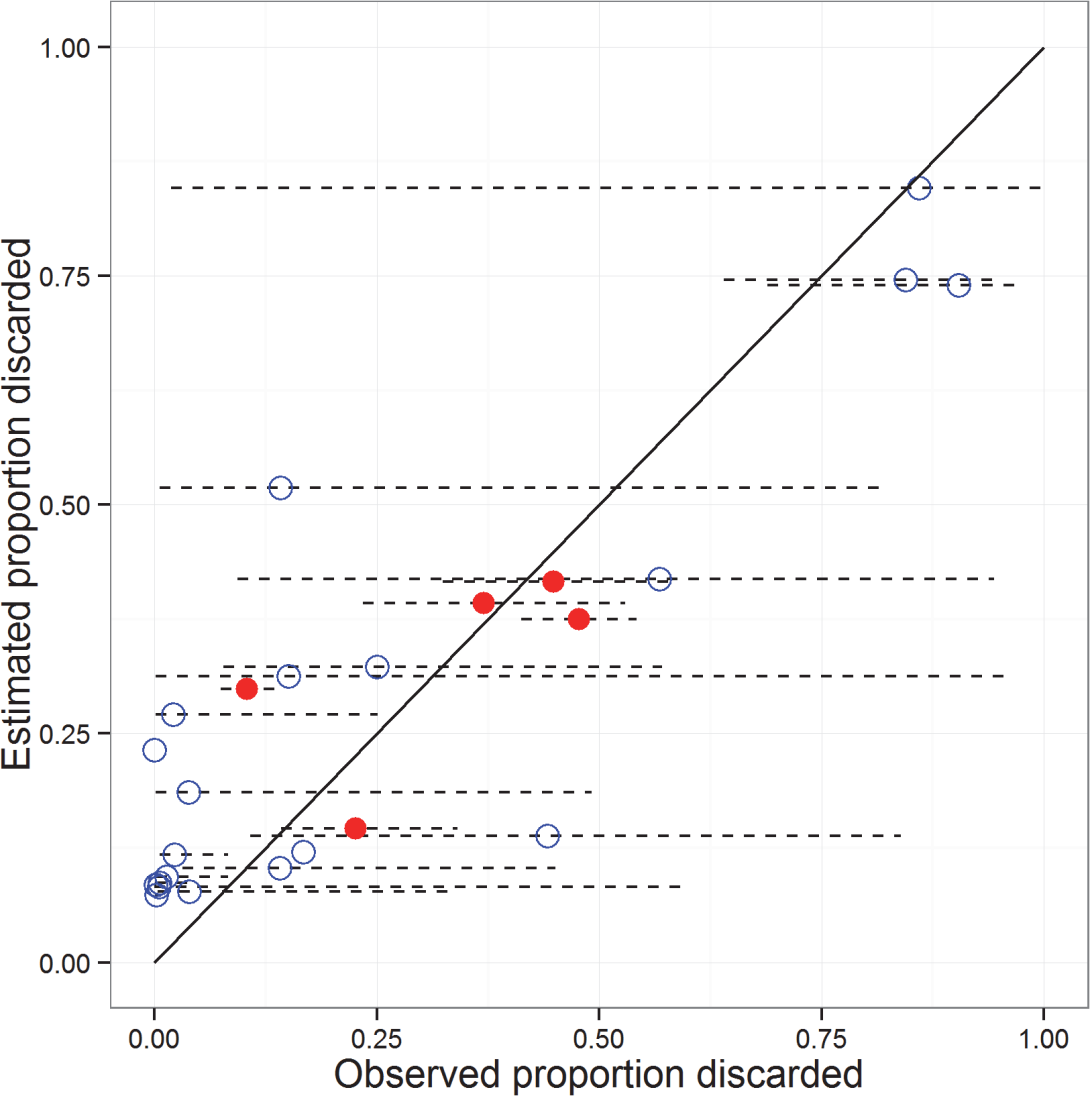
Comparison of modelled and observed proportions discarded. Mean observed proportions of catch discarded from the ICES data in the case of the reference species (averaged over 2001–2010), and from fragments of data harvested from the literature in the case of all other species (S3 Table). Estimated proportion discarded averaged over 2001–2010 from the reduced model in all cases. Horizontal dashed lines indicate ± 1 s.d. of logit transformed observed data. Red circles: reference species; blue circles: all other species. Black line indicates the 1:1 relationship.

We compared the reduced model estimates of proportions of catch discarded for each species (including the reference species) with the fragments of data from those national sampling programmes where information was available (S3 Table; Fig. 10). There is a low likelihood that these observed data are truly representative of the full range of fleets in the North Sea for all species, so they can only be used to provide a rough indication of species discard rates. Compared with these data, the model tended to under-predict the discard rate of heavily discarded species (dab and gurnard) by 10–20%, and over-predict the most lightly discarded species by a similar amount (e.g. sole, ling, hake). This apparent bias for low values of *p* is due the prior assumed for quantity-related discarding, *q*, which was the posterior of the *q* values from the reference species. As there was little information in the data to update this prior, the model estimates reflect discarding practices for the reference species. However, the observed values were generally extremely heterogeneous and the estimated values lay well within the range of values observed.

### Overall quantities and composition of discards

**Fig 11:**
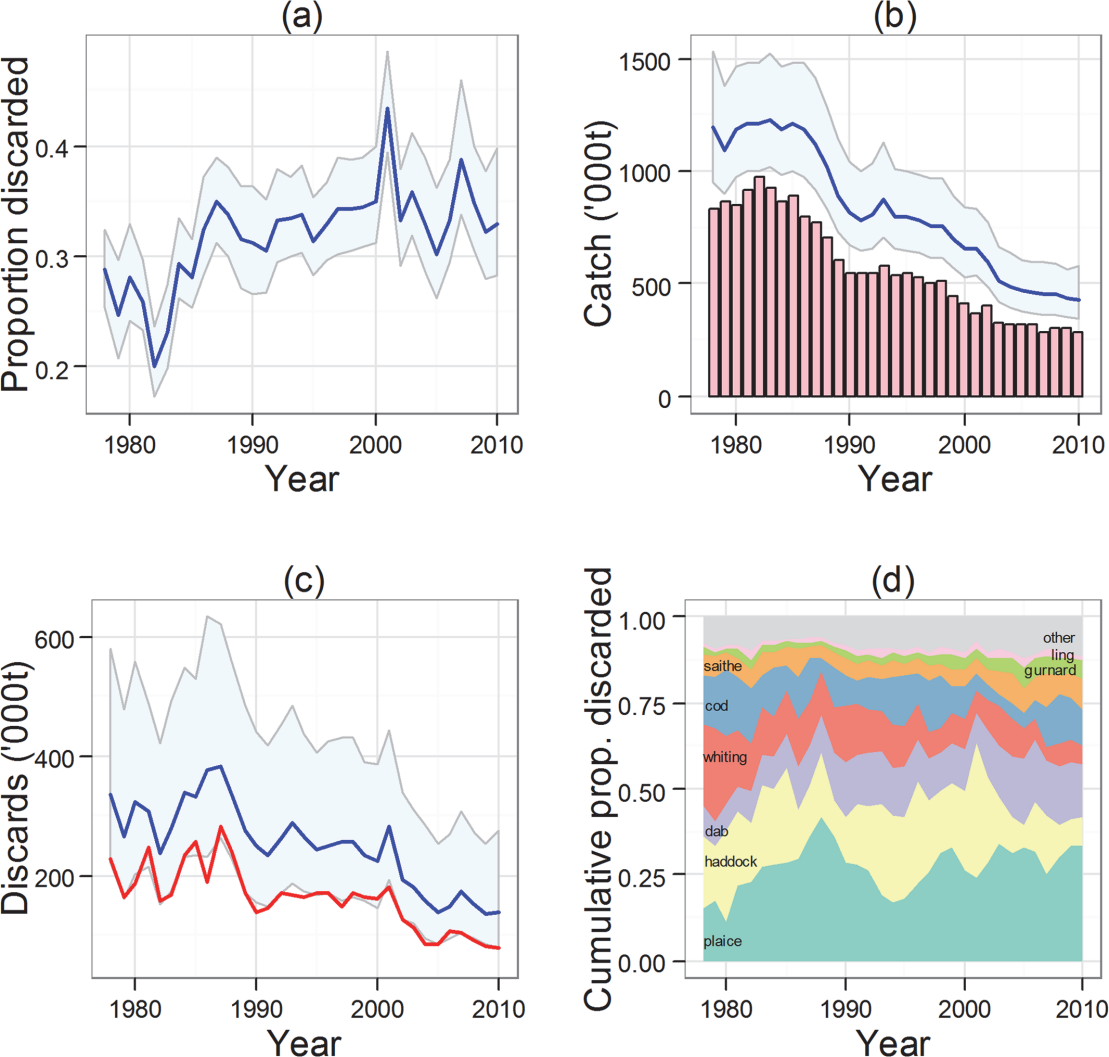
Summary of model results for the entire demersal fish assemblage. (a) Annual proportion of total demersal fish catch discarded (mean and 95% credible interval). (b) Total catch and landings of demersal fish. The blue line and shaded area indicates the mean and 95% credible interval of model estimated total catch, whilst the vertical bars indicate the measured landings. (c) Quantities (thousands of tonnes) of all demersal fish discarded. Blue line and shaded area indicates the mean and 95% credible interval of total discard quantity. The red line indicates the size-related weight of fish discarded, hence the area between the red and blue lines represents quantity-related discards. (d) Annual proportions of the eight most abundant species in the discarded weight of demersal fish (in rank order of long-term average proportions: plaice, haddock, dab, whiting, cod, saithe, gurnard, ling).

Combining the model estimates of discards for all of the species groups with the corresponding data on landings (S7 Table), we estimated the overall annual proportion of demersal fish catch which was discarded. The results (Fig. 11) indicated that overall discarding rate increased from 0.29 in the 1980s to 0.35 in the 2000s. However, the overall quantities discarded decreased from a mean of approximately 300,000 tonnes (95% credible interval 200,000–550,000 tonnes) in the 1980s, to less than 150,000 tonnes in the 2000s (95% credible interval 100,000–350,000 tonnes). Size-related discarding accounted for 60–65% of all discards throughout the period.

Plaice was the top-ranked species in terms of discard quantity throughout the investigation period, with extreme annual discards of over 150,000 tonnes during the 1980s (Fig. 11). A major factor in this regard is that plaice are taken in a fishery targeted at sole which is a smaller but higher value species, and the mesh size is correspondingly small and sub-optimal for larger flatfish. Haddock contributed heavily to discards in years following strong young-of-the-year recruitment events. However, the proportion of discards accounted for by the five reference species declined over time from 0.731 (s.d. 0.026) in the 1980s to 0.584 (s.d. 0.060) on the 2000s.

**Fig 12:**
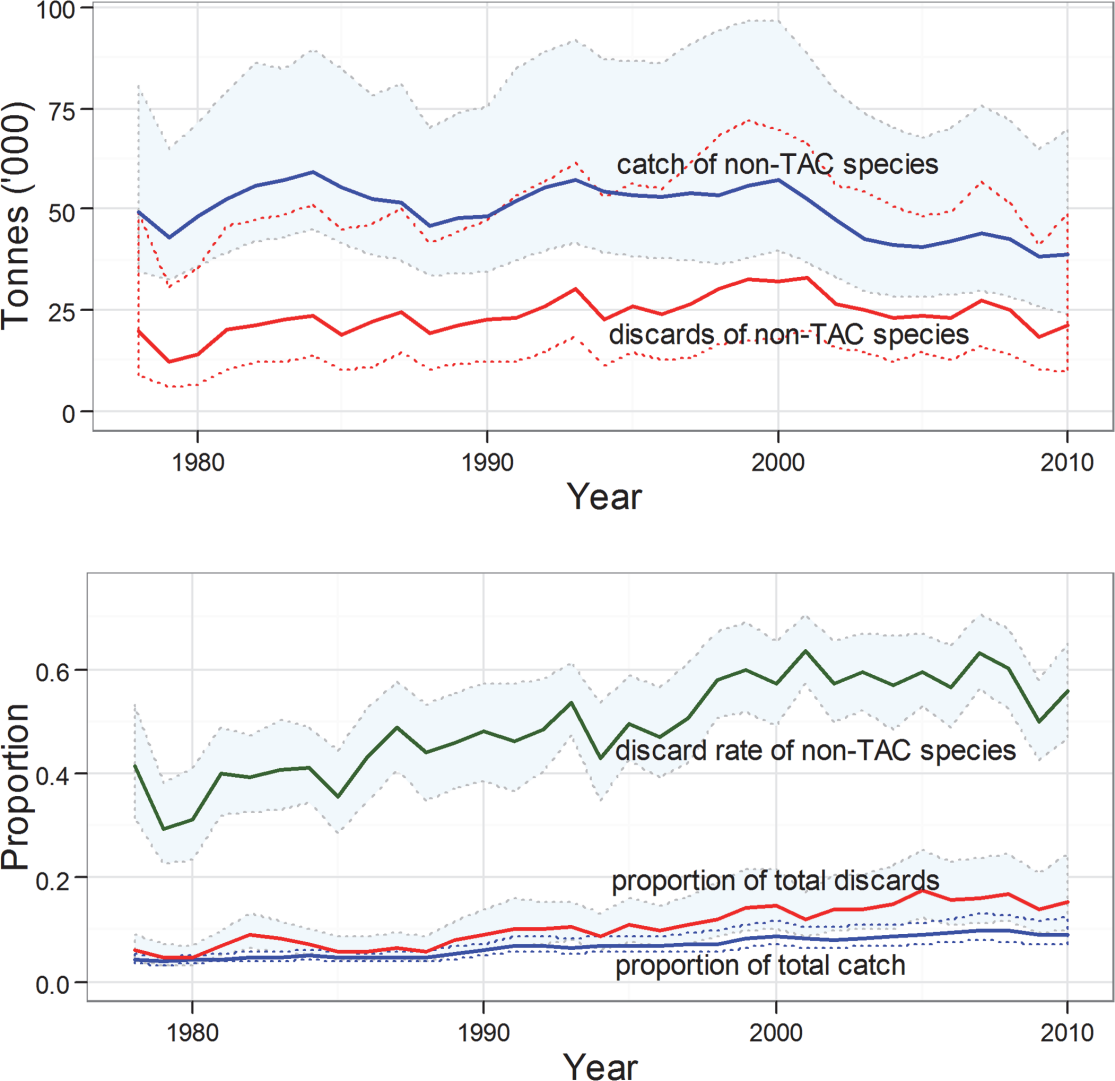
Model results for non-TAC species. (a) Annual catch and discard quantities (tonnes) for the group of species which, in 2010, were not subject to any landing quota (TAC) restrictions (bass, gurnards, halibut, ling, mullets, pollack, tusk, wolfish, and the ‘other marketable’ and ‘discard-only’ groups). (b) catch and discards of non-TAC species as proportions of total demersal catch and discards (solid lines), and the discard rate (proportion of catch discarded) for non-TAC species as a group (dashed line). 95% credible intervals around each of the time series indicated by shaded regions and/or thin lines.

As the proportion of reference species declined in the overall discard quantity, other species increased. In particular, common dab formed an increasing component of discards, along with the group of species which, in 2010, were not subject to any landing quota restrictions (TAC)—bass, gurnards, halibut, ling, mullets, pollack, tusk, wolfish, and the ‘other marketable’ and ‘discard-only’ groups (S8 Table). Catches of these ‘non-TAC’ species were estimated to have decreased by around 10% between the 1980s and 2000s (annual average 53,875 tonnes (s.d. 5,565 tonnes) during the 1980s; 47,676 tonnes (s.d. 6,728 tonnes) during the 2000s; Fig. 12), but this was only a small decrease compared to the ∼60% decline in overall demersal catches. At the same time, our results indicate that discard quantities of these non-TAC species increased by ∼20% (annual average 22,695 tonnes (s.d. 2,950 tonnes) during the 1980s; 27,927 tonnes (s.d. 4,588 tonnes) during the 2000s). Hence, these non-TAC species have comprised an increasing proportion of both the overall demersal catch and discards during the modelled period, and their discard rate has increased from between 0.3 and 0.4 in the 1980s, to a maximum of 0.6 in the 2000s (Fig. 12).

## Discussion

Existing estimates of annual global discards are around 7.3 million tonnes, representing a discard rate of 8% of total catch [3,5]. Rates vary widely between regions and fisheries, and the North Sea was estimated to be a global hot-spot of discarding during the 1980’s and 1990’s, accounting for approximately 13% of the worldwide total (500,000–900,000 tonnes (comprising 65–85% fish and 15–35% benthic invertebrates; [3,30,81]. However, these estimates of total discard quantities are derived by the traditional method of averaging the fragments of discard proportions collected over several decades, and applying the resulting values to landings data in order to estimate the quantity discarded and do not explicitly consider the effect of changing size distribution on discard rates. Our results based on selectivity functions applied to length frequency distributions from research vessel surveys provide new insight on the issue of fisheries discarding in the North Sea, with far greater temporal and taxonomic resolution than has previously been possible.

The overall North Sea annual demersal fish discard weights estimated by our model were consistent with the earlier snapshot values of 320,000–560,000 tonnes during the early 1980’s [30,81], and 330,000 tonnes in 1991 [67], but substantially less than the more recent FAO estimate of 750,0000 tonnes during the period 1992–2001 [5]. Other studies of national fleet discard weights point to the FAO value being an overestimate [82]. However, while the total discarded quantity has declined, the proportion of the catch discarded has tended to increase. Hence, the improvement in quantities discarded appears to be the result of declining catches due both to reduced fishing mortality and declining stock biomass.

The top-ranked discard species in our analysis throughout the entire period was plaice. To this extent the situation is much as it was at the end of the 19^th^ century, when the perception was that undersize plaice were being heavily discarded in the North Sea, and entire UK Parliamentary debates were devoted to the issue of how and whether to introduce legislation to limit the practice [83–85]. Nevertheless, our results reveal a marked shift in the composition of discards over the 30 year investigation period, with reducing importance of the 5 main target species (cod, haddock, whiting, plaice and sole), and increasing importance of non-target flatfish species, especially dab and species which, in 2010, were not governed by any landing quotas (non-TAC species, especially gurnards). The rising proportion of non-TAC species in both catches and discards is of significance since these species will not be subject to any landing obligations in proposed new legislation under the European CFP [22,27].

Data on dab and gurnards have a large influence on estimates of overall discards in our analysis. Observational data indicate that discard rates of both these species are exceptionally high (average >80% of catch). These rates are in the ‘danger zone’ where small uncertainties in the rate have a disproportionately large effect on discard quantities estimated by the traditional method. Our model was less vulnerable to this difficulty, but nevertheless the credible intervals of discard quantities for these species are wide. The sensitivity analysis showed that the discard quantity estimates for dab and gurnard in particular are sensitive to assumptions about retention selectivity parameters. We used a scaled value of the minimum landing size or its nearest equivalent as a proxy, for the length at 50% retention (*RL*_*50*_; eq. 22). Our independent information on effective minimum landing sizes for dab and gurnard came from detailed analyses of catch and landing length distributions for selected national fleets (S5 Table), but nevertheless there is scope for significant under or over-estimation of the discards of these species. Taken as a whole our results present a strong case for increased attention to assessment and sampling of these small body-length, high discard rate species.

Rising abundances of dab and gurnards has been a significant factor in their increasing proportions in the overall quantity of discards. However, this has also been the case for a range of previously rare species include mullet, bass and hake for which the North Sea is at the northern edge of their latitudinal distribution range [86–88]. The increase in hake abundance in the northern North Sea since 2000 presents a particular problem for fishing fleets since the precedents for national shares of regional landing quotas (so-called ‘Relative Stability’ rules [89]) are based on historical catches when hake were distributed further south. This leads to a mis-match between local hake abundance and available quota, potentially leading to extensive quantity-related discarding [88,90].

Using the model, it is possible to distinguish between the size and quantity-related discards. For the reference species, our model-based hind-casts show that most of the discarding can be explained by the size distribution of the catch and that the estimated retention size is consistent with the legal minimum landing size. For these species, quantity-related discarding has clearly not been the main factor determining the overall discard quantities or proportions, at least up to around 2000. Since 2000 there is evidence of large scale quantity-related discarding of both cod and whiting. This is consistent with evidence gathered by observer programmes [91], and has been suggested to be a consequence of the introduction of registration schemes for fish sellers and buyers which curtailed the scope for illegal landing and sale of catch in excess of quotas [91,92]. We also show that the size-related component dominates discards for many other species, notably bass, ling, hake, lemon sole, mullet, skates and rays and other marketable. The majority of quantity-related discards are associated with low value species for which there is only a weak market regardless of specimen size (e.g. dab, gurnards, flounder). For these species we assumed a uniform prior (i.e. no presumption against quantity discarding) which allowed the model to estimate *q* freely. In most cases the mean estimated values of *q* were below prior mean of 0.5 suggesting that there is some information to the data to estimate *q*. Since these species tend to be unrestricted by landing quotas the high rates of quantity-related discards are most probably caused by their low market value making them unattractive to land. These results are of significance because much of the publicity campaign to mobilise opposition to discarding [16] has centred on the proposition that the current fisheries regulatory system established under the EU CFP, which sets annual landing quotas for many fish species, is primarily responsible for the scale of discarding (e.g. Fish Fight [93]). The portrayal is that most fish are discarded to satisfy quota restrictions, whereas our results show that this is not the case at all.

Clearly, our results depend on a number of assumptions in the model. The first and foremost of these is that capture and retention selectivity parameters have remained constant over time. This was forced upon us by the lack of high quality information on regionally integrated length distributions of either catches or landings. Such data are not accessible for any species at the scale of international catches for the whole North Sea and we could only crudely infer such data from the published age compositions of landings and discards. On face value, the assumption of time-independence seems at odds with the changes in legal minimum landing sizes that have been implemented for some species (Table 3), and the considerable research effort and legislative time that has been devoted to devising and implementing technical measures, especially trawl design and mesh size limits, intended to shift capture selectivity towards larger sizes in commercial fisheries. Changes in gear design have certainly led to substantial reductions in the discards in some fisheries, for example, finfish discards in the North Sea shrimp (*Crangon crangon*) fishery [94]. Similarly, trawl fisheries for Norway lobster in the North Sea generate high rates of fish discards due to the small mesh sizes employed, and have been a particular focus for technical measures to reduce undersize fish by-catch [76]. Data on the relative length distributions of whiting in English Nephrops trawls and IBTS data show a noticeable change in capture selectivity coincident with a change in mesh size regulations [82]. Nevertheless, despite such case studies showing improvements in selectivity of particular fleets, there is evidence of poor adherence to fishing gear regulations in general, especially where these lead to negative economic impacts for fishers [95,96]. The close fit to the reference species landings and discard data of our full model (that assumes constant capture selectivity) is an indication that trends in selectivity are hard to detect and are probably small. In addition, evidence from North Sea-wide stock assessments is that relative capture selectivity by age has changed very little over time [59]. It seems likely that variation in the size composition of catches is primarily driven by the size distribution of fish in the sea.

Species capture selectivities vary widely between fishing gears, and sole, in particular, are inefficiently captured by otter trawls. The majority of commercial catches of sole, and also plaice, are taken by beam trawl fisheries, and our estimates of the catch ratio *Q*-parameter indicate that the IBTS Q1 survey is less efficient at sampling the sole stock available to the fleets than for any of the other reference species. This probably explains why our reduced model performed least well for sole in the cross-validation analysis, predicting greater than expected quantity-related discarding for such a high value species. In the cross-validation analysis, the estimated *SL*_50_ was lower, and the *RL*_50_ higher, than in the reference case leading to these large discard rates. Parameter estimates for this species are also more uncertain because there are gaps in the time series of observations. Fortunately, commercial catches of sole, as with a number of other species, although very important in terms of economic value in the North Sea, make only a minor contribution in terms of total discard weight, so the consequences for our overall goal of hind-casting total discards are not severe.

We have based the model on survey data collected during the first quarter of each year, whilst in reality the fisheries operate year-round. Hence the survey catch size distribution is a biased estimate the annual catch size distribution. Due to individual growth, fish represented in the survey by a given size, are caught by fisheries later in the year at some larger size. Hence the survey index is a biased estimate of the size composition of fish available to the fleets, but this infelicity is absorbed into the fitted capture selectivity parameters. Hence, the model fits the reference species data well, and the cross-validation test worked remarkably well for the five reference species.

We have relied on official landings data for the non-reference species from the ICES/FAO database, which do not account for under- or over-reporting of catches. There is ample evidence that official statistics have understated the true total landings at some times, for some species and regions [20,97–99]. For the most part, illegal landings involve the high-value reference species in our model where the gains from capitalising on over-quota catch may be judged to be greater than the risk of detection and ensuing penalty. Hence, when the risk of detection is increased by, for example licensing of fish sellers and buyers, fish that would have been illegally landed are then forced to be discarded [91]. For the reference species we used ICES estimates of mis-reported catches so any bias should be reduced, but for the remaining species our model will under-estimate discard quantities if the official landings are biased low through mis-reporting and vice versa.

Our results have implications for the management of fisheries, and efforts to minimise discarding. The fact that size is the predominant factor affecting discarding, at least for the major targeted species, indicates that management efforts to reduce over-fishing and restore stocks to a state in which they contain a higher proportion of large fish will, in the end, be the most effective remedy for discarding. The proportion by weight of fish larger than minimum landing sizes is analogous to the large fish indicator (LFI; proportion by weight of fish >40 cm; [100]) which has been adopted by the EU Marine Strategy Framework Directive (MSFD) as an indicator of demersal fish community health. The assumption is that the LFI is in some sense inversely related to fishing mortality rates [101]. Reducing fishing mortality is the key management measure envisioned to restore the LFI to target values, and we can expect this to have a commensurate effect on discard rates. Simply prohibiting discarding, without either reducing overall fishing mortality or achieving material improvement in selectivity so that currently discarded fish are no longer caught [14,22,41], seems unlikely to yield any substantial conservation benefits.

Finally, proposed legislation to implement landing obligations for demersal species as part of the EU CFP will only apply to those which are subject to quota restrictions. Observational data on discards of the non-TAC species which will be outside the new legislation are sparse, so their catches are only poorly known. Our results indicate that these species represent a growing proportion of the demersal catch, and that their combined discard rate is already high (∼0.6). Some of these species command high market values (halibut, ling, tusk) creating an incentive to retain and land, whilst others have low value (gurnards, pollack) and already show a high quantity-related discard rate. We might expect these low value species to be increasingly discarded under the proposed new legislation.

## Supporting Information

S1 Appendix OpenBUGS code for the reduced model with an example input data set for lemon sole. The code requires the OpenBUGS 3.2.3 software package for performing Bayesian inference, which is freely available for download at http://www.openbugs.net.
(TXT)

S1 Fig Spatial and temporal distribution of sampling effort during the quarter 1 IBTS surveys, 1978–2011. Individual points in each spatial map indicate a trawl position during the given interval of years. Histogram bars in the bottom right panel indicate the annual total swept area of the trawl surveys.
(PDF)

S2 Fig Biomass densities (kg km^−2^ cm^−1^) of reference species in the quarter 1 IBTS surveys. Colour scale (blue = low, pink = high) scaled to the maximum for each species, so individual panels represent the relative distribution of biomass with respect to time and size. The horizontal line on each panel indicates the legal minimum landing size (MLS).
(PDF)

S3 Fig Biomass densities (kg km^−2^ cm^−1^) of non-reference species in the quarter 1 IBTS surveys. Colour scale (blue = low, pink = high) scaled to the maximum for each species, so individual panels represent the relative distribution of biomass with respect to time and size. The horizontal line on each panel indicates the legal or *de-facto* minimum landing size (MLS).
(PDF)

S4 Fig Time series of the raising factor or catch ratio *Q* for each species. The shaded areas indicate 95% credible intervals around the mean estimates of *Q*. The raising factor *Q* is proportional to the harvest rate for each species.
(PDF)

S5 Fig Weights assigned to reference species catch ratios in order to derive a raising factor for each non-reference species. Values for cod, haddock, whiting, plaice and sole (x-axis: cod, had, whi, ple, sol) are the estimates arising from the cross-validation analysis.
(PDF)

S1 Table Aggregation of taxonomic entities in the ICES DATRAS database recorded as having been caught in the North Sea during the quarter 1 surveys, and taxonomic entities in the ICES/FAO landings database, to the highest common level of resolution. (PDF)

S2 Table Landed and discarded weights (tonnes) of the five reference species (cod, haddock, whiting, plaice and sole) from the North Sea. (XLS)

S3 Table Literature sourced data on discard rates of species other than the reference set—cod, haddock, whiting and plaice—in the North Sea. (PDF)

S4 Table Weight—length relationship parameters used to convert numbers-at-length in ICES International Bottom Trawl Survey (IBTS) data, to weight-at-length. (XLS)

S5 Table Retention length selectivity parameters (eq. 14 in the main paper) derived from data presented in papers and working group reports. (PDF)

S6 Table Coefficients of determination (r^2^) between the median of the full or reduced model output for a given variable, and the corresponding observations to which the model was fitted. (PDF)

S7 Table Annual discarded and landed weights (tonnes) of demersal fish species and groups in the North Sea, 1978–2010. (XLS)

S8 Table History of annual TACs for species in the North Sea, compiled from a range of ICES Annual Advice Books and Working Group reports. (XLS)
