# Supplementary material for: Hind-Casting the Quantity and Composition of Discards by Mixed Demersal Fisheries in the North Sea
Source: PLoS One. 2015 Mar 16;10(3):e0117078. doi: 10.1371/journal.pone.0117078 (PMC4361349; doi:10.1371/journal.pone.0117078)
Supplement: S1 Fig — Individual points in each spatial map indicate a trawl position during the given interval of years. Histogram bars in the bottom right panel indicate the annual total swept area of the trawl surveys. (PDF) [file pone.0117078.s002.pdf]

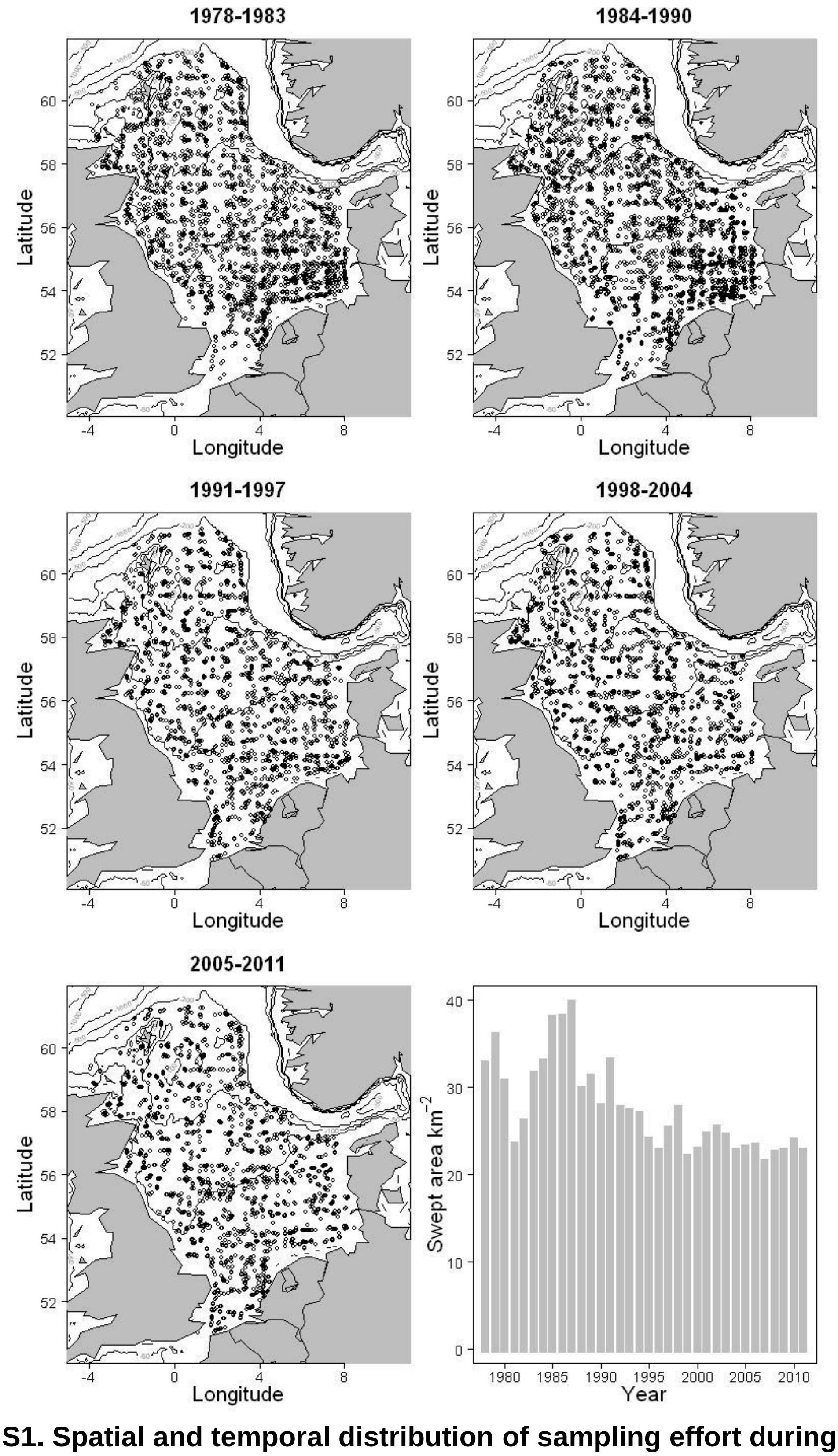

Figure S1. Spatial and temporal distribution of sampling effort during the quarter 1 IBTS surveys, 1978-2011. Individual points in each spatial map indicate a trawl position during the given interval of years. Histogram bars in the bottom right panel indicate the annual total swept area of the trawl surveys.
