# Supplementary material for: Hind-Casting the Quantity and Composition of Discards by Mixed Demersal Fisheries in the North Sea
Source: PLoS One. 2015 Mar 16;10(3):e0117078. doi: 10.1371/journal.pone.0117078 (PMC4361349; doi:10.1371/journal.pone.0117078)
Supplement: S2 Fig — Colour scale (blue = low, pink = high) scaled to the maximum for each species, so individual panels represent the relative distribution of biomass with respect to time and size. The horizontal line on each panel indicates the legal minimum landing size (MLS). (PDF) [file pone.0117078.s003.pdf]

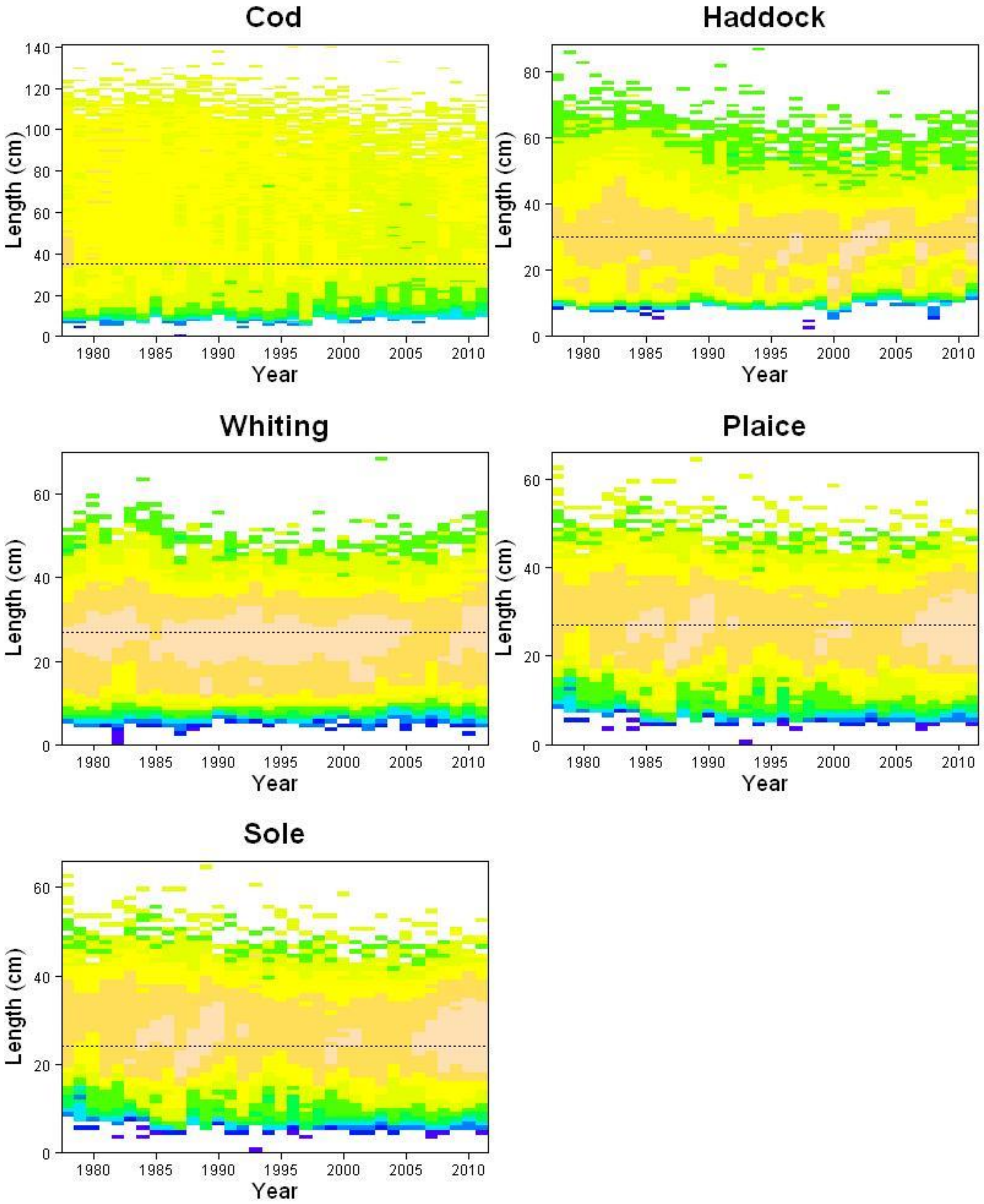

Figure S2. Biomass densities (kg km^{-2} cm^{-1}) of reference species in the quarter 1 IBTS surveys. Color scale (blue = low, pink = high) scaled to the maximum for each species, so individual panels represent the relative distribution of biomass with respect to time and size. The horizontal line on each panel indicates the legal minimum landing size (MLS).
