# Supplementary material for: Hind-Casting the Quantity and Composition of Discards by Mixed Demersal Fisheries in the North Sea
Source: PLoS One. 2015 Mar 16;10(3):e0117078. doi: 10.1371/journal.pone.0117078 (PMC4361349; doi:10.1371/journal.pone.0117078)
Supplement: S4 Fig — The shaded areas indicate 95% credible intervals around the mean estimates of Q. The raising factor Q is proportional to the harvest rate for each species. (PDF) [file pone.0117078.s005.pdf]

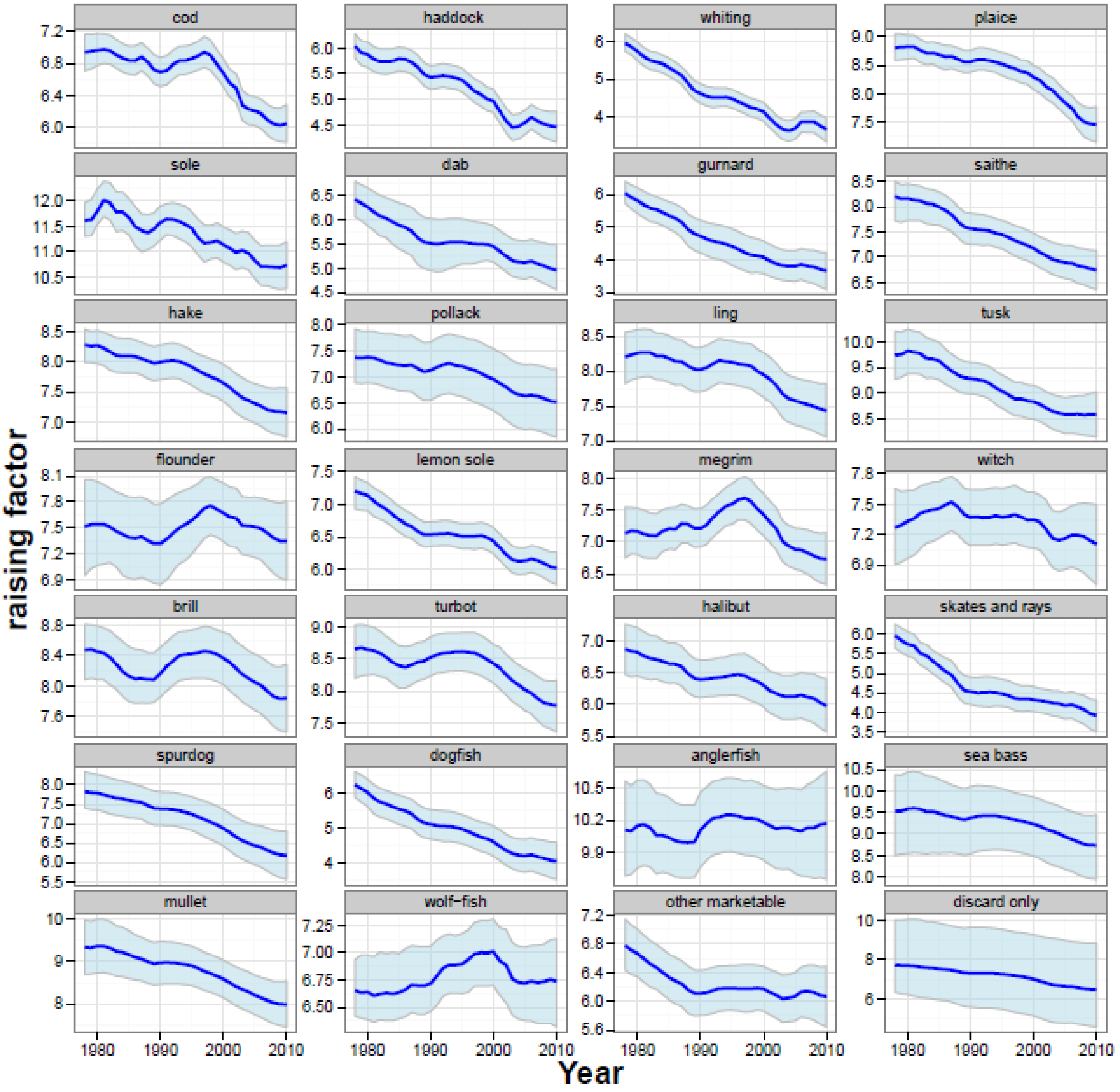

Figure S4. Time series of the raising factor or catch ratio *Q* for each species. The shaded areas indicate 95% credible intervals around the mean estimates of *Q*. The raising factor *Q* is proportional to the harvest rate for each species.
