# Supplementary material for: Hind-Casting the Quantity and Composition of Discards by Mixed Demersal Fisheries in the North Sea
Source: PLoS One. 2015 Mar 16;10(3):e0117078. doi: 10.1371/journal.pone.0117078 (PMC4361349; doi:10.1371/journal.pone.0117078)
Supplement: S5 Fig — Values for cod, haddock, whiting, plaice and sole (x-axis: cod, had, whi, ple, sol) are the estimates arising from the cross-validation analysis. (PDF) [file pone.0117078.s006.pdf]

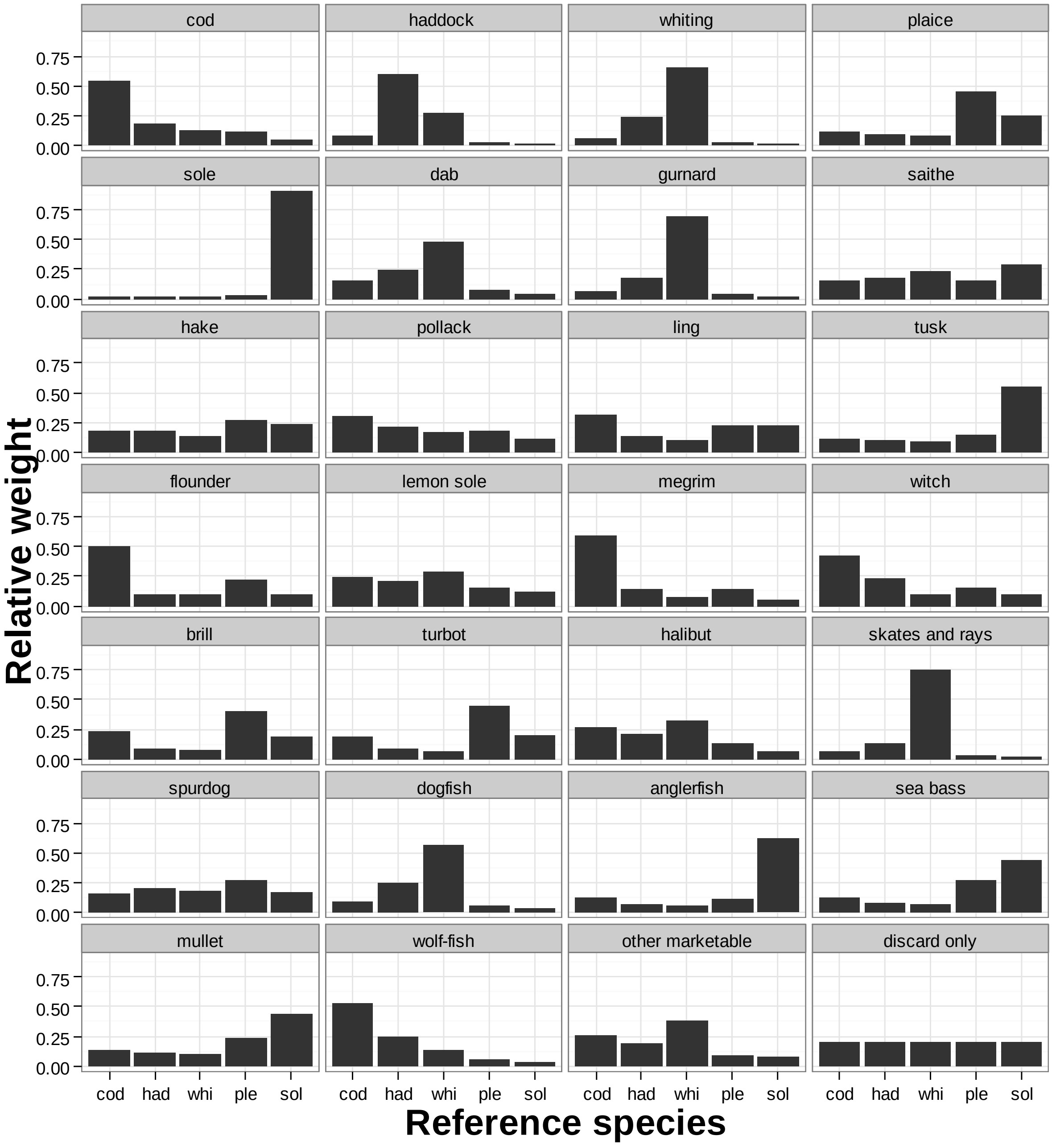

Figure S5. Weights assigned to reference species catch ratios in order to derive a raising factor for each non-reference species. Values for cod, haddock, whiting, plaice and sole (x-axis: cod, had, whi, ple, sol) are the estimates arising from the cross-validation analysis.
