# Supplementary material for: Hind-Casting the Quantity and Composition of Discards by Mixed Demersal Fisheries in the North Sea
Source: PLoS One. 2015 Mar 16;10(3):e0117078. doi: 10.1371/journal.pone.0117078 (PMC4361349; doi:10.1371/journal.pone.0117078)
Supplement: S1 Table — (PDF) [file pone.0117078.s007.pdf]

TABLE S1. Aggregation of taxonomic entities in the ICES DATRAS database [1] recorded as having been caught in the North Sea during the quarter 1 surveys, and taxonomic entities in the ICES/FAO landings database [2], to the highest common level of resolution.

ICES DATRAS taxonomy		FAO/ICES landings taxonomy	Discard model species taxonomy
**ITIS code**	**Scientific / Common name**	**Common name**	**Common name**
164712	*Gadus morhua* - Atlantic cod	Atlantic cod	Cod
164744	*Melanogrammus aeglefinus* – Haddock	Haddock	Haddock
164758	*Merlangius merlangus* – Whiting	Whiting	Whiting
172902	*Pleuronectes platessa* -European plaice	European plaice	Plaice
164727	*Pollachius virens* –Saithe	Saithe(=Pollock)	Saithe
172980	Solidae- Soles	Common sole	Sole
173000	*Solea* sp. – Sole	Soles	
173001	*Solea vulgaris* – Common sole		
173002	*Solea solea* – Common sole		
164760	*Molva molva* – Ling	Ling	Ling
164789	Merlucciidae – Hakes	European hake	Hake
164795	*Merluccius merluccius* – Hake		
172835	*Lepidorhombus whiffiagonis* – Megrim	Megrim	Megrim
172834	*Lepidorhombus boscii* – Fourspot megrim		
164497	Lophidae – Anglerfishes	Angler(=Monk)	Anglerfish
164501	*Lophius piscatorius* - Anglerfish	Monkfishes	
164502	*Lophius budegassa* – Blackbellied anglerfish	Anglerfishes	
164728	*Pollachius pollachius* – Pollack	Pollack	Pollack
170315	Percichthyidae – Basses	European seabass	Bass
170316	*Dicentrarchus* sp. Bass	Seabasses	

170317	*Dicentrarchus labrax* – Seabass	Striped bass	
		Spotted seabass	
164740	*Brosme brosme* – Tusk	Tusk	Tusk
172881	*Limanda limanda* - Common dab	Common dab	Dab
172894	*Platichtys flesus* - European flounder	European flounder	Flounder
172714	Bothidae - Lefteye flounders	Lefteye flounders	
172806	*Arnoglossus imperialis* – Lefteye flounder		
172888	*Microstomus kitt* - Lemon sole	Lemon sole	Lenon sole
172873	*Glyptocephalus cynoglossus* - Witch	Witch flounder	Witch
		Righteye flounders	
		Winter flounder	
616195	*Psetta maxima* – Turbot	Turbot	Turbot
172749	*Scophthalmus rhombus* – Brill	Brill	Brill
160617	*Squalus acanthis* – Spurdog	Spurdog	Spurdog
160838	*Torpedo marmorata* – Electric ray	Rays and skates	Rays and skates
160845	Rajidae – Rays	Blue skate	
160846	*Raja* sp. – Ocellate rays	Cuckoo ray	
160880	*Raja brachyuran* – Blonde ray	Longnosed skate	
160882	*Raja microcellata* – Painted ray	Rays and skates	
160883	*Raja montagui* – Spotted ray	Sandy ray	
160900	*Raja undulate* – Undulate ray	Shagreen ray	
160901	*Raja clavata* – Thornback ray	Small-eyed ray	
160959	*Dasyatis pastinaca* – Common stingray	Spotted ray	
564126	*Dipturus batis* – Blue skate	Thornback ray	
564128	*Leucoraja circularis* – Cuckoo ray	White skate	
564134	*Leucoraja fullonica* – Shagreen ray	Rays, stingrays, mantas	

564140	*Leucoraja lentiginosa* – Speckled skate	Blonde ray	
564141	*Dipturus linteus* – Sail ray	Arctic skate	
564143	*Leucoraja naevus* – Cuckoo ray	Common stingray	
564148	*Dipturus oxyrinchus* – Longnosed skate	Starry ray	
564149	*Amblyraja radiate* – Starry skate	Undulate ray	
		Sharks, rays, skates, etc.	
159911	*Lamna nasus* – Porbeagle	Cartilaginous fishes	Dogfishes and sharks
159985	Scyliorhinidae – Catsharks	Dogfishes and hounds	
160034	*Galeus melastomus* - Blackmouth catshark	Blue shark	
160053	*Scyliorhinus* sp. – Spotted catsharks	Catsharks, nursehounds	
160065	*Scyliorhinus canicila* – Spotted catshark	Porbeagle	
160067	*Scyliorhinus stellaris* – Nursehound	Tope shark	
160181	*Galeorhinus galeus* – Tope	Various sharks	
160226	*Mustelus* sp. - Smooth-hounds	Dogfish sharks	
160240	*Mustelus asterias* – Starry smooth-hound	Dogfish sharks, etc	
160242	*Mustelus mustelus* –Smooth-hound	Black dogfish	
160604	Squalidae – Spiny dogfioshes	Lanternsharks	
		Leafscale gulper shark	
		Nursehound	
		Portuguese dogfish	
		Small-spotted catshark	
		Greenland shark	
		Catsharks, etc.	
		Shortfin mako	
		Birdbeak dogfish	
		Gulper shark	

		Sailfin roughshark	
		Shortnose velvet dogfish	
		Dogfishes	
		Smooth-hounds	
		Smooth-hound	
		Angelshark	
		Dusky shark	
		Tiger shark	
		Houndsharks,smoothhounds	
		Thresher sharks	
169418	*Mullus surmeletus* – Red mullet	Mullets	Mullets
169419	*Mullus barbatus* – Striped mullet	Red mullet	
170333	Mugilidae – Grey mullets	Surmullets(=Red mullets)	
170335	*Mugil cephalus* – Grey mullet	Thicklip grey mullet	
170371	*Chelon labrosus* – Thicklip mullet	Boxlip mullet	
170377	*Liza aurata* – Golden mullet		
171335	Anarhichadidae – Wolffishes	Atlantic wolfish	Wolffish
171336	*Anarhichas* sp. - Wolfish	Wolffishes(=Catfishes)	
171341	*Anarhichas lupus* – Atlantic wolfish	Northern wolfish	
171342	*Anarhichas minor* – Spotted wolfish	Spotted wolfish	
171345	*Anarhichthys ocellatus* – Northern wolfish	Sea catfishes	
172933	*Hippoglossus hippoglossus* - Halibut	Atlantic halibut	Halibut
		Greenland halibut	
692071	*Chelidonichthys cuculus* – Red gurnard	Grey gurnard	Gurnards
166972	Triglidae – Grurnards	Red gurnard	
167044	*Eutrigla gurnardus* – Grey gurnard	Tub gurnard	

167046	*Trogloporus lastoviza* – Streaked gurnard	Gurnards, searobins	
643890	*Chelidonichthys lucernus* – Smallscaled gurnard	Gurnards	
		Streaked gurnard	
160670	*Etmopterus spinax* - Velvet belly	Amer. plaice(=Long rough dab)	Minor marketable
161022	*Chimaera monstrosa* - Rabbitfish	Arctic char	
161125	Anguillidae - Freshwater eels	Artic cod	
161128	*Anguilla anguilla* - European eel	Atlantic gobies nei	
161341	*Conger conger* - Conger eel	Atlantic searobins	
162315	*Alepocephalus bairdii* - Baird's smoothhead	Atlantic thornyhead	
164457	Gobiesocidae - Goby sp.	Axillary seabream	
164475	*Lepadogaster* sp. - Goby sp.	Baird's slickhead	
164482	*Diplecogaster bimaculata* - Clingfish	Ballan wrasse	
164751	*Phycis blennoides* - Greater forkbeard	Black cardinal fish	
164754	*Trisopterus minutus* - Poor cod	Black scabbardfish	
164755	*Trisopterus luscus* - Bib	Black seabream	
164764	*Gaidropsarus* sp. - Three-bearded rocklings	Blackbelly rosefish	
164765	*Gaidropsarus vulgaris* - Three-bearded rockling	Blackspot(=red) seabream	
164766	*Gaidropsarus mediterraneus* - Shore rockling	Blue ling	
164761	*Molva dypterygia* – Blue ling	Burbot	
164772	*Gadiculus argenteus* - Silvery pout	Common carp	
165419	*Trachyrincus murrayi* - Roughnose grenadier	Common mora	
166025	*Atherina presbyter* - Common sand smelt	Corkwing wrasse	
166287	*Zeus faber* - European john dory	Crucian carp	
166704	Scorpaenidae - Scorpionfishes	Cyprinids	
166756	*Sebastes mentella* - Deepwater redfish	Eelpout	
166779	*Sebastes viviparus* - Redfish	European conger	

166787	*Helicolenus dactylopterus* - Blackbelly rosefish	European eel
166839	*Scorpaena scrofa* - Bigscale scorpionfish	European perch
167188	*Icelus bicornis* - Twohorn sculpin	European whitefish
167210	*Artediellus atlanticus* - Atlantic hookear sculpin	Freshwater bream
167311	*Myoxocephalus* sp. - Shorthorn sculpins	Freshwater breams
167316	*Myoxocephalus quadricornis* - Fourhorn sculpin	Gilthead seabream
167317	*Myoxocephalus scorpioides* - Arctic sculpin	Goldsinny-wrasse
167318	*Myoxocephalus scorpius* - Shorthorn sculpin	Greater forkbeard
167483	Cyclopteridae - Lumpfishes	Greater weever
167612	*Cyclopterus lumpus* - Lumpfish	Groupers, seabasses
167749	*Epinephelus acanthistius* - Gulf coney	John Dory
167862	*Serranus scriba* - Lettered perch	Large-eyed rabbitfish
169180	Sparidae - Porgies	Lumpfish(=Lumpsucker)
169207	*Pagrus pagrus* - Red porgy	Moras
169215	*Pagellus erythrinus* - Porgie	Mugil spp
169229	*Spondyliosoma cantharus* - Black seabream	Northern pike
170733	*Ctenolabrus rupestris* - Goldsinny wrasse	Offshore rockfish
170737	*Labrus bergylta* - Ballan wrasse	Orange roughy
170739	*Labrus mixtus* - Cuckoo wrasse	Patagonian toothfish
170992	*Trachinus draco* - Greater weever	Pike-perch
171746	Gobiidae - Goby	Polar cod
171833	*Gobius* sp. - Goby	Poor cod
171841	*Gobius cobitis* - Giant goby	Porgies, seabreams
171850	*Gobius niger* - Black goby	Pouting(=Bib)
171854	*Gobius paganellus* - Rock goby	Rabbit fish
171859	*Gobius gasteveni* - Goby	Ratfishes

171971	*Crystallogobius linearis* - Crystal goby	Red codling
171977	*Pomatoschistus* sp. - Goby	Red porgy
171978	*Pomatoschistus minutus* - Freckled goby	Roach
171980	*Pomatoschistus pictus* - Painted goby	Rocklings
171982	*Pomatoschistus microps* - Common goby	Roughhead grenadier
172033	*Aphia minuta* - Transparent goby	Roundnose grenadier
172036	*Lesueurigobius friesii* - Fries' goby	Sabanejewia balcanica
172877	*Hippoglossoides platessoides* - American dab	Sand smelt
173022	*Microchirus* sp. - Thickback sole	Sand sole
173026	*Microchirus variegatus* - Thickback sole	Scorpionfishes nei
173051	*Pegusa lascaris* - Sand sole	Scorpionfishes, rockfishes
201978	*Gasterosteus aculeatus* - Threespined stickleback	Sculpins
630409	*Echiichthys vipera* - Lesser weever	Senegalese sole
636752	*Buenia jeffreysii* - Jeffrey's goby	Silver scabbardfish
637881	*Pomatoschistus lozanoi* - Goby	Silvery pout
644150	*Micrenophrys lilljeborgii* - Scaleless sculpin	Spiny vase
644643	*Triglops pingelii* - Ribbed sculpin	Sticklebacks
		Sturgeons
		Tench
		Thickback sole
		Thickback soles
		Triggerfishes, durgons
		Velvet belly
		Weeverfishes nei
		Weevers nei
		White hake

		Wrasses, hogfishes, etc.	
		Wreckfish	
		Yellowedge grouper	
159700	*Lampetra* sp. – Lamprey		Discard-only
159719	*Lampetra fluviatilis* - Lamprey		
159721	*Petromyzon* sp. - Lamprey		
159722	*Petromyzon marinus* - Sea lamprey		
159772	*Myxine glutinosa* - Atlantic hagfish		
164748	*Enchelyopus cimbrius* - Fourbeard rockling		
164768	*Gaidropsarus argentatus* - Arctic threebearded rockling		
164777	*Raniceps raninus* - Lesser forkbeard		
164780	*Ciliata septentrionalis* - Northern rockling		
164846	*Ophidion barbatum* - Snake blenny		
165116	*Echiodon drummondii* - Pearlfish		
165215	Zoarcidae - Blennies		
165255	*Lycodes* sp. - Viviparous blenny		
165284	*Lycodes vahlii* - Checker eelpout		
165324	*Zoarces viviparus* - Viviporous blenny		
165612	*Scomberesox saurus* - Atlantic saury		
166363	Gasterosteidae - Sticklebacks		
166365	*Gasterosteus aculeatus* - Alaskan stickleback		
166401	*Spinachia spinachia* - Fifteenspine stickleback		
166438	Syngnathoidei - Pipefishes		
166443	Syngnathidae - Pipefishes		
166444	*Syngnathus* sp.- Seaweed pipefishes		
166463	*Syngnathus rostellatus* - Lesser pipefish		

166464	*Syngnathus acus* - Great pipefish
166467	*Syngnathus typhle* - Broadnosed pipefish
166595	*Nerophis ophidion* - Straightnosed pipefish
166613	*Acentronura* sp. - Pipefishes
167196	Cottidae - Bullheads
167375	*Triglops murrayi* - Moustache sculpin
167390	*Taurulus bubalis* - Longspined sea scorpion
167408	*Cottunculus microps* - Arctic sculpin
167454	*Agonus cataphractus* - Armed bullhead
167478	*Leptagonus decagonus* - Atlantic poacher
167550	*Liparis* sp. - Sea snails
167578	*Liparis liparis* - Striped seasnail
167581	*Liparis montagui* - Montagu's seasnail
170297	*Pterycombus brama* - Atlantic fanfish
171124	Blenniidae - Blennies
171125	*Blennius* sp. - Blennies
171554	Stichaeidae - Shannies
171571	*Chirolophis ascanii* - Atlantic warbonnet
171603	*Leptoclinus maculatus* - Daubed shanny
171645	*Pholis gunnellus* - Rock gunnel
171691	Callionymidae - Dragonets
171692	*Callionymus* sp. - Dragonet
171698	*Callionymus lyra* - Common dragonet
171699	*Callionymus maculatus* - Spotted dragonet
171712	*Callionymus reticulatus* - Dragonet
172803	*Arnoglossus* sp. - Scaldfishes

172805	*Arnoglossus laterna* - Scaldfish		
172809	*Arnoglossus thori* - Grohmann's scaldfish		
172828	*Zeugopterus* sp. - Topknot		
172829	*Zeugopterus punctatus* - Common topknot		
173020	*Buglossidium* sp. - Solenette		
173021	*Buglossidium luteum* - Solenette		
551497	*Macroramphosus scolopax* - Longspine snipefish		
555704	Phycinae - Phycine hakes		
616605	*Zeugopterus regius* - Bloch's topknot		
616613	*Zeugopterus norvegicus* - Norwegian topknot		
623023	*Ciliata mustela* - Fivebeard rockling		
623025	*Gaidropsarus macrophthalmus* - Bigeye rockling		
631023	*Lumpenus lampretaeformis* - Serpent blenny		
631033	*Lycenchelys sarsii* - Sars' wolf eel		
631144	*Lycodes gracilis* -Viviparous blenny		
636467	*Parablennius gattorugine* - Tompot blenny		
644927	*Entelurus aequoreus* - Snake pipefish		
650172	*Sander lucioperca* – Pikeperch		
161700	Clupeidae – Herring/Sprat	Albacore	Major targeted pelagic
161722	*Clupea harengus* - Atlantic herring	Atlantic herring	
161789	*Sprattus sprattus* -European sprat	Atlantic horse mackerel	
164756	*Trisopterus esmarkii* - Norway pout	Atlantic mackerel	
164774	*Micromesistius poutassou*- Blue whiting	Blue whiting(=Poutassou)	
168588	*Trachurus trachurus* - Horse mackerel	Capelin	
171670	Ammodytidae - Sandeels	Clupeoids nei	
171671	*Ammodytes* sp. - Sandeels	European sprat	

171676	*Ammodytes tobianus* - Lesser sandeel	Jack and horse mackerels	
171677	*Ammodytes marinus* - Lesser sandeel	Northern bluefin tuna	
171680	*Gymnammodytes semisquamatus* - Smoothed sandeel	Norway pout	
171681	*Hyperoplus* sp. - Greater sandeel	Sandeels(=Sandlances)	
171682	*Hyperoplus lanceolatus* - Greater sand eel	Striped marlin	
171683	*Hyperoplus immaculatus* - Corbin's sand eel	Swordfish	
172414	*Scomber scombrus* - Atlantic mackerel	Tuna-like fishes	
161813	*Sardina pilchardus* - European pilchard	European anchovy	Minor targeted pelagic
161831	*Engraulis encrasicolus* - European anchovy	European pilchard(=Sardine)	
		Sardinellas	
161701	*Alosa* sp. - Shads	Alfonsinos	By catch pelagic
161708	*Alosa alosa* - Allis shad	Allis and twaite shads	
161994	*Salmo* sp. - Salmonids	Argentine	
161996	*Salmo salar* - Atlantic salmon	Argentines	
161997	*Salmo trutta* - Brown trout	Atlantic pomfret	
162039	*Osmerus eperlanus* - Smelt	Atlantic redfishes	
162057	Argentinidae - Argentines	Atlantic salmon	
162061	*Argentina* sp. - Argentines	Barracudas nei	
162064	*Argentina silus* - Atlantic argentine	Beaked redfish	
162071	*Argentina sphyraena* - Silver smelt	Bogue	
165594	*Belone belone* - Garfish	Boarfish	
166271	Zeiformes – Dories	Chars nei	
166284	*Zenopsis conchifera* - American john dory	Common pandora	
166309	Caproidae - Boarfishes	Diadromous fishes	
166320	*Capros aper* - Boarfish	European smelt	
166756	*Sebastes mentella* - Deepwater redfish	Garfish	

166779	*Sebastes viviparous* - Redfish	Golden redfish	
170290	*Brama brama* - Atlantic pomfret	Greater argentine	
173414	*Mola mola* - Ocean sunfish	Rainbow trout	
572694	*Alosa agone* – Shad	Salmonoids nei	
615903	Lampridae – Opah	Sea trout	
644687	*Arctozenus risso* – White barracudina	Shads	
		Silversides(=Sand smelts)	
		Sunfish	
		Trouts nei	
		Twaite shad	
		Vendace	
162187	*Maurolicus muelleri* - Pearlside		Discard–only pelagic
162368	Myctophiformes – Myctophids		

The right-hand column shows the corresponding identities used to estimate discard quantities with the model. ITIS code [3] (left-hand column) refers to the species identity code used in the ICES DATRAS database.

References for Table S1

1. ICES (2010) Report of the International Bottom Trawl Survey Working Group (IBTSWG). ICES CM 2010/SSGESST:06, 261 pp. ICES DATRAS data centre (http://www.ices.dk/marine-data/data-portals/Pages/DATRAS.aspx)
2. Lassen H, Cross D, Christiansen E (2012) One hundred years of catch statistics for the Northeast Atlantic. ICES Community Research Report 311. 25pp.
3. Interagency Taxonomic Information System. http://www.itis.gov/
