# Supplementary material for: Hind-Casting the Quantity and Composition of Discards by Mixed Demersal Fisheries in the North Sea
Source: PLoS One. 2015 Mar 16;10(3):e0117078. doi: 10.1371/journal.pone.0117078 (PMC4361349; doi:10.1371/journal.pone.0117078)
Supplement: S3 Table — (PDF) [file pone.0117078.s009.pdf]

TABLE S3. Literature sourced data on discard rates of species other than the reference set --cod, haddock, whiting and plaice - in the North Sea.

Species	Years	Proportion discarded by weight	Source	Reference
Anglerfish	1999 – 2007	0.140	England & Wales TRIO observer database	[1]
Anglerfish	2010	0.003	Denmark, North Sea & Skagerrak, observer data	[2]
Anglerfish	2005-2007	0.021	Summary for beam trawl fisheries	[3]
Anglerfish	2002-2008	0.003 (otter trawl)	Germany, observer data	[4]
Bass	2000’s	0	Summary by WG	[5]
Brill	2010	0.007	Denmark, North Sea & Skagerrak, observer data	[2]
Brill	2005-2007	0.012	Summary for beam trawl fisheries	[3]
Brill	2002-2008	0.019 (beam trawl); 0.141 (otter trawl)	Germany, observer data	[4]
Common dab	1999 – 2007	0.969	England & Wales TRIO observer database	[1]
Common dab	2007, 2008	0.96, 0.95	Netherlands observer database	[6]
Common dab	2007-2009	0.67, 0.95, 0.97 (otter trawl); 0.99, 0.96, 0.95 (beam trawl)	Germany, observer data, otter trawl and beam trawl	[5]
Common dab	2003 – 2006	0.89 (by numbers)	England & Wales observer data	[7]
Common dab	2010	0.285	Denmark, North Sea & Skagerrak, observer data	[2]
Common dab	1990’s	0.60-0.70	Irish observer data from beam trawl fisheries	[8, 5]
Common dab	2005-2007	0.838	Summary for beam trawl fisheries	[3]
Common dab	2008-2008	0.866 (beam trawl); 0.594-0.818 (otter trawl depending on cod-end mesh)	Germany, observer data	[4]
Common sole	1999 – 2007	0	England & Wales TRIO observer database	[1]
Common sole	1976-79, 1980-1983, 1989-1990, 1999, 2000, 2001, 2002, 2003, 2004, 2005, 2006, 2007, 2008	0.04, 0.15, 0.20, 0.05. 0.10, 0.06, 0.13, 0.14, 0.17, 0.11, 0.13, 0.10, 0.06	Netherlands observer database	[6]
Common sole	1978, 1979, 1980, 1981, 1982, 1983, 1989, 1990, 1999, 2000, 2001, 2002, 2003, 2004, 2005, 2006, 2007, 2008	0.03, 0.03, 0.09, 0.09, 0.09, 0.09, 0.13, 0.11, 0.08, 0.08, 0.08, 0.13, 0.14, 0.17, 0.11, 0.13, 0.10, 0.06	Netherlands beam trawl fishery data compiled for ICES Assessment WG	[9]
Common sole	2003 – 2006	0.20 (by numbers)	England & Wales observer data	[7]
Common sole	2010	0.275	Denmark, North Sea, observer data	[2]

Common sole	2005-2007	0.122	Summary for beam trawl fisheries	[3]
Common sole	2002-2008	0.076 (beam trawl); 0.058 (otter trawl)	Germany, observer data	[4]
Flounder	1999 – 2007	0.954	England & Wales TRIO observer database	[1]
Flounder	2010	0	Denmark, North Sea & Skagerrak, observer data	[2]
Flounder	2005-2007	0.448	Summary for beam trawl fisheries	[3]
Flounder	2002-2008	0.119 (beam trawl)	Germany, observer data	[4]
Gurnards	1999 – 2007	0.899	England & Wales TRIO observer database	[1]
Gurnards	1980's – 2000's	>0.95	Summary by WG	[5]
Gurnards	2003 – 2006	0.74 (by numbers)	England & Wales observer data	[7]
Gurnards	2010	0.911	Denmark, North Sea & Skaggerak, observer data	[2]
Gurnards	2005-2007	0.976 (grey gurnard); 0.632 (red gurnard)	Summary for beam trawl fisheries	[3]
Gurnards	2002-2008	0.364 (tub gurnard, beam); 0.295 (tub gurnard, otter trawl); 0.930 (grey gurnard, beam trawl); 0.766-0.863 (grey gurnard, otter trawl depending on cod end mesh)	Germany, observer data	[4]
Hake	1999 – 2007	0.220	England & Wales TRIO observer database	[1]
Hake	2010	0.182	Denmark, North Sea & Skagerrak, observer data	[2]
Hake	2002-2008	0 - 0.002 (otter trawl, depending on mesh size)	Germany, observer data	[4]
Halibut	1999 – 2007	0	England & Wales TRIO observer database	[1]
Halibut	2010	0.219	Denmark, North Sea & Skagerrak, observer data	[2]
Lemon sole	1999 – 2007	0.293	England & Wales TRIO observer database	[7]
Lemon sole	2003 – 2006	0.39 (by numbers)	England & Wales observer data	[7]
Lemon sole	2010	0.041	Denmark, North Sea & Skagerrak, observer data	[2]
Lemon sole	2005-2007	0.164	Summary for beam trawl fisheries	[3]
Lemon sole	2002-2008	0.732 (beam trawl); 0.025-0.347 (otter trawl depending on cod end mesh)	Germany, observer data	[4]
Ling	2010	0.13	Denmark, North Sea & Skagerrak, observer data	[2]
Ling	2002-2008	0.003	Germany, observer data	[4]
Megrim	1999 – 2007	0.339	England & Wales TRIO observer database	[1]
Megrim	2006-2008	0.05	Scottish data, northern North Sea	[10]
Mullets	2000's	0	Summary by WG	[5]
Mullets	2010	0	Denmark, North Sea & Skagerrak, observer data	[2]

Pollack	2010	0.003	Denmark, North Sea & Skagerrak, observer data	[2]
Pollack	2002-2008	0.001 (otter trawl)	Germany, observer data	[4]
Rays and skates	1999 – 2007	0.025	England & Wales TRIO observer database	[1]
Rays and skates	2003 – 2006	0.51 – 0.56 (by numbers)	England & Wales observer data	[7]
Rays and skates	2010	0	Denmark, North Sea & Skagerrak, observer data	[2]
Rays and skates	2002-2008	0.743 (beam trawl); 09,21-0.985 (otter trawl depending on cod end mesh)	Germany, observer data	[4]
Saithe	1999 – 2007	0.328	England & Wales TRIO observer database	[1]
Saithe	2003 – 2006	0.28 (by numbers)	England & Wales observer data	[7]
Saithe	2010	0.021	Denmark, North Sea & Skagerrak, observer data	[2]
Saithe	2002-2008	0.001 (otter trawl)	Germany, observer data	[4]
Saithe	2009-2011	<0.05 (otter trawl)	French and German fleets in the northern North Sea	[11]
Small sharks	1999 – 2007	0	England & Wales TRIO observer database	[1]
Spurdog	1999 – 2007	0.015	England & Wales TRIO observer database	[1]
Spurdog	2010	0.64	Denmark, North Sea & Skagerrak, observer data	[2]
Turbot	1999 – 2007	0.167 (turbot and brill combined)	England & Wales TRIO observer database	[1]
Turbot	1980's – 2000's	0.024 (turbot and brill combined)	Summary by WG	[5]
Turbot	2010	0	Denmark, North Sea & Skagerrak, observer data	[2]
Turbot	2005-2007	0.005	Summary for beam trawl fisheries	[3]
Turbot	2002-2008	0.013 (beam trawl); 0-0.02 (otter trawl depending on cod end mesh)	Germany, observer data	[4]
Tusk	2010	0	Denmark, North Sea & Skagerrak, observer data	[2]
Witch	1999 – 2007	0.750	England & Wales TRIO observer database	[1]
Witch	2010	0.173	Denmark, North Sea & Skagerrak, observer data	[2]
Wolffish	1999 – 2007	0	England & Wales TRIO observer database	[1]
Wolffish	2010	0.002	Denmark, North Sea & Skagerrak, observer data	[2]
Wolffish	2002-2008	0.001	Germany, observer data	[4]
Other gadoids	1999 – 2007	0.059	England & Wales TRIO observer database	[1]
Other demersal fish	1999 – 2007	0.007	England & Wales TRIO observer database	[1]
Other demersal fish	2003 – 2006	0.66 (by numbers)	England & Wales observer data	[7]
Other marketable	2010	0.944	Grenadier, Long rough dab, Lumpfish, Rabbitfish, Rocklings, Velvet belly, Weevers	[2]
Other marketable	1999 – 2007	1	Long rough dab	[1]

Other marketable	2002-2008	0.973 (bib, long rough dab, solenette)	Germany, observer data	[4]
Major targeted pelagic	2010	0.991	Herring, Horse mackerel, Mackerel, Blue whiting, Norway pout, Sprat	[2]
Major targeted pelagic	1999-2007	0.535	Herring, Horse mackerel, Mackerel, Blue whiting, Norway pout, sandeels, Sprat	[1]
Major targeted pelagic	2002-2008	0.715 (Mackerel, Horse mackerel)	Germany, observer data	[4]
Minor targeted pelagic	2010	1	European anchovy	[2]
By-catch pelagic	2010	0.971	Argentines, Redfish	[2]
Discard only	2010	1	Hooknose	[2]
Discard only	1999 – 2007	1	Dragonet	[1]

Data for common sole are also listed even though this is a reference species, since the ICES approved data on discard rates are based entirely on observer data from the Netherlands observer programme, whilst other less extensive data exist. Proportions discarded are either averages over a period of years where a single value is given, or annual values where multiple figures are listed.

References for Table S3

1. Mackinson S, Daskalov G (2007) An ecosystem model of the North Sea to support an ecosystem approach to fisheries management: description and parameterisation. *Scientific Series Technical Report* 142, Cefas Lowestoft, 195pp.
2. Storr-Paulsen M, Håkansson KB, Egekvist J, Degel H, Dalskov J (2012) Danish sampling of commercial fishery. Overview with special attention to discards 2010 data. DTU Aqua Report 250-2012, 86pp.
3. European Commission (2011) Studies in the Field of the Common Fisheries Policy and Maritime Affairs. Lot 4: Impact Assessment Studies related to the CFP. Impact Assessment of Discard Reducing Policies. EU Discards Annex. Project no: ZF0926_S10. 71pp.
4. Ulleweit J, Stransky C, Panten K (2010) Discards and discarding practices in German fisheries in the North Sea and Northeast Atlantic during 2008-2008. Journal of Applied Ichthyology 26: 54-66.
5. ICES (2010) Report of the Working Group on Assessment of New MoU species (WGNEW). ICES Advisory Committee, ICES CM 2010/ACOM:21, 603pp.
6. Rőckmann C, Quirijns F, van Overzee H, Uhlmann S (2011). Discards in fisheries – a summary of three decades of research at IMARES and LEI. IMARES Report C068/11, 41pp.
7. Enever R, Revill AS, Grant A (2009) Discarding in the North Sea and on the historical efficacy of gear-based technical measures in reducing discards. Fisheries Research 95: 40–46.
8. Borges L, Rogan E, Officer R (2005) Discarding by the demersal fishery in the waters around Ireland. Fisheries Research 76: 1-13.

9. ICES (2012) Report of the Working Group on the Assessment of Demersal Stocks in the North Sea and Skagerrak (WGNSSK). ICES Advisory Committee, ICES CM 2012/ACOM:13, 1383pp. - North Sea sole assessment data p555-608
10. ICES (2012) Report of the Working Group for the Celtic Seas Ecoregion (WGCSE). ICES Advisory Committee, ICES CM 2012/ACOM:12, 1750pp.
11. ICES (2012) Report of the Working Group on the Assessment of Demersal Stocks in the North Sea and Skagerrak (WGNSSK). ICES Advisory Committee, ICES CM 2012/ACOM:13, 1383pp. – North Sea saithe assessment data p609-670
