# Supplementary material for: Hind-Casting the Quantity and Composition of Discards by Mixed Demersal Fisheries in the North Sea
Source: PLoS One. 2015 Mar 16;10(3):e0117078. doi: 10.1371/journal.pone.0117078 (PMC4361349; doi:10.1371/journal.pone.0117078)
Supplement: S5 Table — (PDF) [file pone.0117078.s011.pdf]

TABLE S5. Retention length selectivity parameters (eq. 14 in the main paper) derived from data presented in papers and working group reports.

Species	Area & year	Gear	Retention selectivity parameters RL_{50}, RI	Literature source
Cod	North Sea, 2005	Otter trawl	38, 17	[1]
Cod	North Sea, 2005	Beam trawl	35, 3	[1]
Cod	NE Atlantic, 2005	Otter trawl	33, 6	[1]
Cod	NE Atlantic, 2005	Pair trawl	33, 6	[1]
Cod	NE Atlantic, 2005	Seine	38, 11	[1]
Haddock	North Sea, 2005	Otter trawl	32, 7	[1]
Haddock	NE Atlantic, 2005	Otter trawl	32, 11	[1]
Haddock	NE Atlantic, 2005	Pair trawl	33, 10	[1]
Haddock	NE Atlantic, 2005	Seine	32, 11	[1]
Hake	North Sea, 2005	Otter trawl	33, 8	[1]
Plaice	North Sea, 2005	Otter trawl	27, 5	[1]
Plaice	North Sea, 2005	Beam trawl	26, 4	[1]
Saithe	NE Atlantic, 2005	Otter trawl	44, 18	[1]
Saithe	NE Atlantic, 2005	Pair trawl	48, 15	[1]
Saithe	NE Atlantic, 2005	Seine	54, 18	[1]
Sole	North Sea, 2005	Otter trawl	29, 16	[1]
Sole	North Sea, 2005	Beam trawl	24, 7	[1]
Whiting	North Sea, 2005	Otter trawl	27, 5	[1]
Whiting	NE Atlantic, 2005	Otter trawl, Pair trawl, seine	32, 13	[1]
Whiting	North Sea	Nephrops Trawl	25, 6	[2]
Grey gurnard	Irish Sea, 2005	Otter trawl	30, 6.6	[3]
Grey gurnard	Celtic Sea, 2005	Otter trawl	36, 2.2	[3]
Tub gurnard	English Channel, 2005	Otter trawl	25, 3.3	[3]
Dab	North Sea, 2007	Otter trawl	25.5, 3.3	[3]
Dab	North Sea, 2008	Otter trawl	24.5, 0.55	[3]
Dab	North Sea, 2009	Otter trawl	26.5, 0.88	[3]
Dab	North Sea, 2007	Beam trawl	24.0, 1.0	[3]
Dab	North Sea, 2008	Beam trawl	23.5, 1.5	[3]
Dab	North Sea, 2009	Beam trawl	25, 1.9	[3]
Turbot	Irish & Celtic Sea, 2005	Beam trawl	29.5, 0.4	[3]
Brill	Irish & Celtic Sea, 2005	Beam trawl	29.5, 1.8	[3]

RL_{50} refers to the length at 50% retention aboard fishing vessels; RI refers to the selection interval.

References for Table S5

1. European Commission (2006) Commission staff working paper report of the Scientific, Technical, and Economic Committee for Fisheries. Discards from community vessels: opinion expressed during a plenary meeting held in Ispra from 6–10 November 2006. 56 pp. http://stecf.jrc.ec.europa.eu/reports.
2. Catchpole TL, Frid CLJ, Gray TS (2006) Resolving the discard problem — A case study of the English *Nephrops* fishery. Marine Policy 30: 821–831.
3. ICES (2010) Report of the Working Group on Assessment of New MoU species (WGNEW). ICES Advisory Committee, ICES CM 2010/ACOM:21, 603pp.
