# Supplementary material for: Hind-Casting the Quantity and Composition of Discards by Mixed Demersal Fisheries in the North Sea
Source: PLoS One. 2015 Mar 16;10(3):e0117078. doi: 10.1371/journal.pone.0117078 (PMC4361349; doi:10.1371/journal.pone.0117078)
Supplement: S6 Table — (PDF) [file pone.0117078.s012.pdf]

Table S6. Coefficients of determination (r^2) between the median of the full or reduced model output for a given variable, and the corresponding observations to which the model was fitted.

Model	Species	Landings	Biomass index	Discards
Full	**cod**	0.9953	0.9527	0.9654
Full	**haddock**	0.9777	0.7224	0.7486
Full	**whiting**	0.9763	0.5204	0.8489
Full	**plaice**	0.9972	0.6554	0.6134
Full	**sole**	0.9928	0.5535	-0.3361
Reduced	**cod**	0.9973	0.9462	0.2023
Reduced	**haddock**	0.9893	0.6747	0.4705
Reduced	**whiting**	0.9961	0.5689	0.5582
Reduced	**plaice**	0.9982	0.6047	0.1416
Reduced	**sole**	0.9904	0.3993	-0.0613
Reduced	dab	0.9982	0.8536	NA
Reduced	gurnard	0.9989	0.7596	NA
Reduced	saithe	0.9931	0.4301	NA
Reduced	hake	0.9972	0.5875	NA
Reduced	pollack	0.9649	-0.0008	NA
Reduced	ling	0.8662	0.0074	NA
Reduced	tusk	0.9751	0.1845	NA
Reduced	flounder	0.9983	0.2154	NA
Reduced	lemon sole	0.9934	0.6333	NA
Reduced	megrim	0.9842	0.2470	NA
Reduced	witch	0.9914	0.3636	NA
Reduced	brill	0.9931	0.0129	NA
Reduced	turbot	0.9785	0.1649	NA
Reduced	halibut	0.9959	0.4096	NA
Reduced	skates and rays	0.9907	0.4896	NA
Reduced	spurdog	0.9999	0.3023	NA
Reduced	dogfish	0.9889	0.5749	NA
Reduced	anglerfish	-0.0377	0.7183	NA
Reduced	sea bass	0.6530	0.4287	NA
Reduced	mullet	0.7823	0.8943	NA
Reduced	wolf-fish	0.9991	0.7796	NA
Reduced	other marketable	-0.6240	0.9067	NA
Reduced	discard only	NA	0.8503	NA

NA indicates that there were no observed data with which to compare the model output.
Reference species indicated in bold.
